# *Physella acuta* Confirmed as Intermediate Host of *Posthodiplostomum* sp. from Lake Alqueva, Portugal

**DOI:** 10.3390/pathogens14040304

**Published:** 2025-03-23

**Authors:** Maria Teresa Bispo, Isabel Larguinho Maurício, Pedro Manuel Ferreira, Silvana Belo, Manuela Calado

**Affiliations:** Global Health and Tropical Medicine (GHTM), LA-REAL, Instituto de Higiene e Medicina Tropical (IHMT), Universidade NOVA de Lisboa, Rua da Junqueira 100, 1349-008 Lisboa, Portugal; isabel.mauricio@ihmt.unl.pt (I.L.M.); pedroferreira@ihmt.unl.pt (P.M.F.); silvanabelo@ihmt.unl.pt (S.B.)

**Keywords:** trematodiasis, *Posthodiplostomum* sp., *Physella acuta*, Lake Alqueva, Portugal

## Abstract

*Physella acuta* is an invasive freshwater snail with a global distribution and a recognized role as an intermediate host for various trematodes, including *Echinostoma* spp. and *Trichobilharzia physellae*. In Portugal, *P. acuta* is commonly found in freshwater bodies such as Lake Alqueva, the largest artificial reservoir in Europe. The lake’s creation has altered local ecosystems, influencing freshwater snail populations and migratory bird activity, which may contribute to the dispersal of trematode parasites. While *P. acuta* is present in the region, its role in trematode transmission remains unclear. This study investigated *P. acuta* as a potential intermediate host for trematodes in Lake Alqueva. Freshwater snails were collected from 18 sites, with cercarial shedding induced under artificial light. Infected snails were found in 2 of the 18 snail populations surveyed. A sequence analysis of the amplified ITS2 rDNA region confirmed the presence of *Posthodiplostomum* sp., implicating, for the first time, *P. acuta* as an intermediate host for this parasite in Portugal. This study highlights the need for further research on *P. acuta*’s role in trematode transmission and potential impact on local ecosystems to assess parasitic risks to veterinary and public health.

## 1. Introduction

Aquatic environments serve as key transmission sites for parasitic trematodes, including those of medical and veterinary importance, which require freshwater snails as intermediate hosts to complete their life cycle [1]. The presence of these snail hosts directly influences the distribution and persistence of these parasites in freshwater ecosystems [2].

The snail family Physidae comprises freshwater pulmonated gastropods with a broad geographic distribution, and some species are intermediate hosts for several trematodes, including avian schistosomes, those responsible for cercarial dermatitis, and fish trematodes [3,4,5]. *Physella* (syn. *Physa*) *acuta* (Draparnaud, 1805), commonly known as the bladder snail, is the most widespread species of this family [6]. It has a global distribution and is well known for its invasive potential [7]. This species thrives in diverse aquatic environments, and its success as an invasive species is attributed to its rapid reproductive cycle, tolerance to environmental fluctuations, and strong resistance to pollutants and infections, demonstrating exceptional adaptability [8,9]. Human activities, including aquaculture and the trade of aquatic plants, as well as migratory birds have contributed to its dispersal [9,10]. The presence of *P. acuta* raises concerns due to its role as an intermediate host for trematodes like *Echinosthoma* spp. and *Ochetosoma* spp., particularly in regions where its ecological interactions remain understudied [4,11]. Additionally, studies indicate that *P. acuta* exhibits remarkable immunological plasticity, enabling it to tolerate cytotoxic stress and resist infections or even adapt to infection with newly encountered digenetic trematodes [8,11,12,13].

In Portugal, *P. acuta* is widespread, reaching high numbers in various habitats such as rivers, lakes, and artificial reservoirs [7,14]. However, its role as an intermediate host of trematodes within the country remains largely unknown. *P. acuta* may serve as a host for emerging parasitic infections, particularly in response to climate change, habitat modifications, and the introduction of new parasites through bird migration, leading to the establishment of previously undocumented trematodiases in Portugal.

A key location for the study of these host-parasite interactions is Lake Alqueva, the largest artificial water reservoir in Europe, located in southeastern Portugal within the districts of Évora and Beja (UTM 29S). Since its creation in 2002, the lake has driven substantial environmental changes in the region, including habitat transformation, water quality shifts, and altered ecological interactions [15]. These changes have facilitated the establishment and expansion of fish and bird populations, further influenced by climate change, which continues to shape the region’s biodiversity dynamics [15]. As Lake Alqueva increasingly attracts human activity and sustains diverse wildlife, understanding the interactions among invasive snails, trematode parasites, and environmental change is crucial for assessing potential public health and ecological risks. The aim of this work was to assess the potential of *P. acuta* as intermediate host of trematodes, particularly those of human, veterinary, or economic importance.

## 2. Materials and Methods

**Figure 1:**
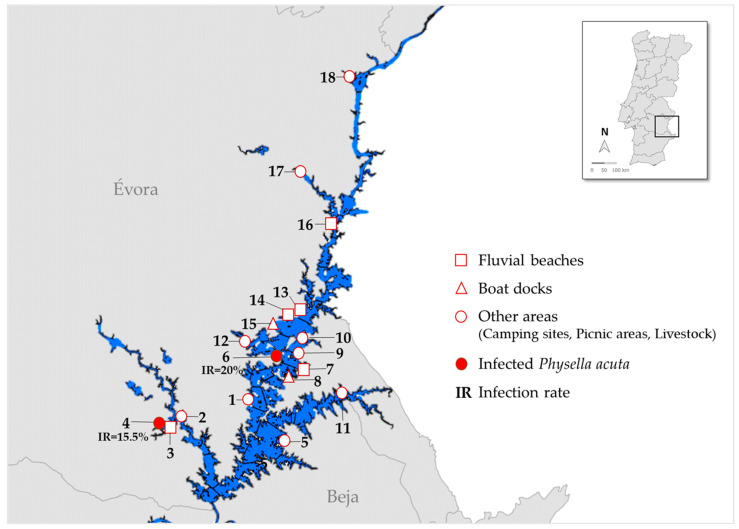
Map of Lake Alqueva indicating the sampling sites and the infected areas with their infection rates.

A malacological survey was conducted at 18 locations around Lake Alqueva in August 2023 (Figure 1) as part of the Alqueva Project, a yearlong exploratory study on cercarial dermatitis. Snails were collected manually by two to three people for 15 min each along the lake’s shoreline, near beaches, boat pontoons, and other areas with access to the water by humans or livestock. Live snails were transported, at ambient temperature in plastic containers with water collected from the same location, to the laboratory at Instituto de Higiene e Medicina Tropical (IHMT). The snails were identified using standard morphological criteria [16,17]. To stimulate cercarial shedding, pools of 10 *P. acuta* individuals were placed in a beaker with 30 mL of dechlorinated water under artificial light for 2 h, after which the water was examined with the aid of a stereomicroscope to detect live cercariae. The procedure was repeated for individual snails from pools with detected cercarial shedding.

The cercariae were identified based on morphological characteristics using classification keys [18,19]. Cercariae from each snail were concentrated by centrifugation at 2040× *g* for 2 min and preserved in 70% ethanol. For molecular identification, genomic DNA was extracted using a CTAB-chloroform protocol adapted from Stothard et al. [20], with modifications. Briefly, ethanol-preserved samples were first centrifuged at 13,800× *g* for 5 min to remove excess ethanol. The supernatant was discarded, and the pellet was dried at room temperature for 10 min. To each sample, 600 µL CTAB buffer at 60 °C and 6 µL Proteinase K (20 mg/mL) were added. The samples were macerated in 1.5 mL tubes using plastic pestles until a homogeneous suspension was obtained and were incubated at 65 °C for 1.5 h, with agitation, or overnight at 36 °C. After digestion, 600 µL of chloroform-isoamyl alcohol (24:1) was added and gently inverted for 2 min. The samples were centrifuged at 13,800× *g* for 1 min. The upper aqueous phase was carefully transferred to a new tube, then 1.0 µL absolute ethanol was added and centrifuged at 13,800× *g* for 15 min. The supernatant was discarded, and the pellet was washed with 500 µL 70% ethanol and centrifuged at 13,800× *g* for 10 min. The final pellet was air-dried at room temperature, and the DNA was resuspended in 50–100 µL TE buffer and stored at 4 °C.

The Ribosomal Internal Transcribed Spacer 1 and 2 (ITS1 and ITS2) regions were amplified by PCR using primers BD1 and 4S [21] and primers ITS2-F and ITS2-R [22], respectively, in a Biometra Tone 96G thermal cycler (Analytik, Jena, Germany). The mitochondrial Cytochrome C Oxidase subunit 1 (COI) was amplified by PCR using universal primers JB3 and JB4 [23]. Each 30 µL PCR reaction contained Supreme NZYTaq II 2x Green Master Mix (NZYTech, Lisboa, Portugal), 30 pmol of each primer, and 3.0 μL of genomic DNA [5–7 ng]. The thermal profiles for each primer set are described in Table 1. The PCR products were visualized under UV light after electrophoresis on a 1.5% agarose gel in TAE buffer stained with ethidium bromide.

The amplified products were Sanger-sequenced commercially using the same primers. The sequences were manually checked and edited in Chromas 2.6.6 (Technelysium, South Brisbane, Australia). For comparison with other available sequences, a BLAST search was performed (https://blast.ncbi.nlm.nih.gov/Blast.cgi), and the 80 closest sequences were downloaded. Outgroup sequences were obtained from a separate BLAST search. The complete sequence data set was aligned using ClustalW as implemented in BioEdit 7.2.5 (Carlsbad, CA, USA) [23]. A phylogenetic analysis was performed using the Neighbor-Joining (NJ) algorithm, with the Tamura-Nei 93 model, in MEGA 11 [24]. A bootstrap analysis with 1000 replicates was performed to check the robustness of the trees. A NeighborNet analysis was also performed in SplitsTree 6 using the Kimura 2-parameter model. 

## 3. Results

In August 2023, a total of 1304 snails identified as *Physella acuta* were collected from 18 locations around Lake Alqueva (Table 2). The average number of collected snails was 72.4 (minimum 1 and maximum 173), with a median of 62.5. The number of snails varied across the sampling sites, likely influenced by environmental factors. Further studies will analyze bioecological data to better understand these variations.

**Figure 2:**
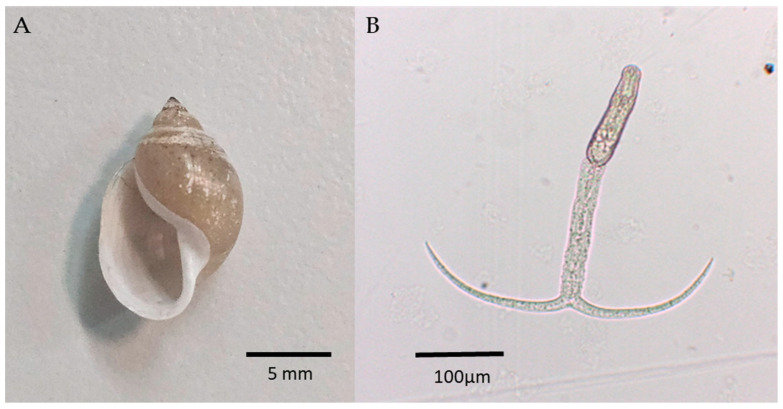
Snail and cercariae samples. (**A**) *P. acuta* specimen from Amieira, Alentejo, Portugal. (**B**) Bifurcated cercaria released from *P. acuta*, visualized under 40× optical microscope.

Two populations of *P. acuta* from two locations shed cercariae upon exposure to light: 15.5% (20/129) from Amieira (38°17′23.04″ N 7°34′0.70″ W) and 20% (2/10) from Mourão (38°20′32.02″ N 7°17′9.00″ W). The overall infection rate was 1.7%. All cercariae released from *P. acuta* were indistinguishable morphologically (Figure 2).

ITS and COI amplification products obtained from four selected samples—Al-C5 (Amieira) and Al-C8, Al-C12, and Al-C13 (Mourão)—were sequenced (Table 3). Upon BLAST analysis, the sequence of the COI product from Al-C8 revealed 95% homology with sequences of the genus *Posthodiplostomum*. The sequence of the ITS1 product from Al-C12 had over 95% homology with *Posthodiplostomum centrarchid*, while the ITS2 sequences with the best quality (Al-C5, Al-C8, and Al-C13) exhibited 99% homology with *Posthodiplostomum* cf. *minimum*. An alignment was produced in BioEdit 7.2.5 (Carlsbad, CA, USA) [24] with the sequences obtained from the BLAST search for COI and for ITS1 regions (Supplementary Information), using only unique sequences. The best mutation model was found to be General Time Reversible with a Gamma distribution (parameter 0.67) and a transition/transversion ratio of 1.88, assuming that a certain fraction of sites are evolutionarily invariable. A Neighbor-Joining (NJ) tree produced in MEGA 11 [25] (Figures S1 and S2) and a NeighborNet network produced by SplitsTree6 (Figure S3) showed that the sequences clustered with *Posthodiplostomum* sp. isolates (COI: OK314916, OK314909, OK314910, OK314917) and *Posthodiplostomum* cf. *minimum* isolate (ITS1: MF171009), albeit in a separate branch to other groups within this genus.

## 4. Discussion

Trematode cercariae released from four *P. acuta* individuals collected from two locations on the margins of the artificial Lake Alqueva, southeastern Portugal, were identified by direct Sanger sequencing of their COI, ITS1, and ITS2 PCR amplification products as belonging to the genus *Posthodiplostomum* (Dubois, 1936). To our knowledge, this is the first known detection of this parasite genus in snails in Portugal, although it had previously been detected in fish [26]. Phylogenetically, these parasites clustered with the genus *Posthodiplostomum* with robust bootstrap support, although in a separate early branch from the other lineages, with no recombination detected with those other lineages. Further research is necessary to determine whether the parasites found within this work belong to a separate *Posthodiplostomum* species.

The genus *Posthodiplostomum*, a diplostomid, has piscivorous birds as its definitive hosts [27] and fish (mostly cyprinids) as its second intermediate hosts [28]. This life cycle makes this genus highly relevant in pisciculture [28]. Cercariae penetrate fish scales and encyst in the skin as metacercariae, leading to melanization around the cyst [29,30]. This process gives rise to the name “black spot” disease, a condition that can significantly impact fish health in the wild as well as in aquaculture [28]. Its negative impacts include weight loss, reduced growth, and impaired ability to evade predators, often resulting in the exclusion of affected fish from commercial markets [30,31]. The geographic distribution of *Posthodiplostomum* sp. is extensive, with reports of infections in freshwater fish across several parts of the world [26]. *Posthodiplostomum* sp. has been identified in numerous water bodies across Europe [26,32], and this first detection of *Posthodiplostomum* in snails in Portugal extends the known range of this parasite genus on this continent.

The identified European trematode species include *Posthodiplostomum cuticola* (Croatia, Czech Republic, Italy, France, Germany, and Poland), *Posthodiplostomum brevicaudatum* (Czech Republic, Russia, and Ukraine), *Posthodiplostomum centrarchi* (Bulgaria, Czech Republic, Germany, Portugal, Slovakia, and Ukraine), and *Posthodiplostomum minimum* (France, Italy, Hungary, and Spain) [26,30,33,34,35]. These species are often associated with cyprinid and centrarchid fish and have *Planorbis planorbis* and *Planorbarius corneus* as their snail intermediate hosts [30,35]. However, studies on the prevalence, host specificity, and ecological impacts of *Posthodiplostomum* sp. in Southern Europe are still limited. The confirmation of the parasite’s presence in this study will contribute to understanding its adaptation and extent in European ecosystems.

Previously, Kvach et al. [26] identified *Posthodiplostomum* in cyprinid fish in Portugal, in the Sado and Tejo rivers. Kvach et al. [36] implicated aquaculture in the spread of the parasite, suggesting that the transport of largemouth black bass (*Micropterus salmoides*) from France contributed to the introduction of this parasite into Ukraine, which had a strain indistinguishable from that found in Portugal [26,36]. Our molecular and phylogenetic analyses provide robust results, enabling the identification of the parasite at the genus level. However, the observed differences, although minor, across the analyzed genetic regions highlight important questions regarding the parasite’s lineage and its precise identification at the species or subspecies level. Although *P.* cf. *minimum centrarchi* was already identified in Portugal [26], these variations may have been influenced by population dynamics or environmental factors, warranting further investigation. Climate change and new routes for migratory birds may also have played a role in introducing new strains of parasites to the Lake Alqueva region, potentially influencing the observed patterns of parasite distribution and transmission.

While *P. acuta* was already known as the first intermediate host for *Posthodiplostomum* sp. in America, its role in Europe was previously unknown [26]. This study helps fill that gap by identifying *P. acuta* as an intermediate host and providing additional molecular confirmation of the presence of the parasite in Portugal. The identification of the first intermediate host of this parasite in the country is a significant step toward understanding the biology of *P. acuta* and its potential adaptability to digenetic trematodes [13,26].

In conclusion, even though *Posthodiplostomum* sp. is not known to be zoonotic, its presence underscores broader “One Health” implications, emphasizing the intricate interconnections among aquatic ecosystems, wildlife, and human activities. This work provides the first molecular evidence that *P. acuta* serves as an intermediate host for *Posthodiplostomum* sp. in Portugal, within Lake Alqueva. In intensive aquaculture systems, where fish are kept at high densities, the spread of parasitic infections can be rapid and devastating. Consequently, the presence of this parasite in Lake Alqueva poses a significant threat to local fish farming. It is crucial to consider the possibility that the birds acting as definitive hosts are migratory, as this may facilitate the spread of *Posthodiplostomum* between different bodies of water along their migratory routes, as studies have shown for other trematodes. These findings highlight the importance of regular malacological surveillance and molecular monitoring to detect and manage potential threats to biodiversity, fishery resources, and ecosystem services in freshwater environments to control the spread of the parasite.

## Figures and Tables

**Table 1:** Thermal profile for PCR amplifications.

Region	Primer Name	Primer Sequence	Amplification Thermal Profile
ITS1 [21]	BD1	5′-GTCGTAACAAGGTTTCCGTA-3′	95 °C—5 min
	4S	5′-TCTAGATGCGTTCGAARTGTCGATG-3′	(35 cycles) 95 °C—1 min, 60 °C—1 min and 72 °C—1 min; 72 °C—5 min
ITS2 [22]	ITS2F	5′-CTT GAA CGC ACA TTG CGG CCA TGG G-3′	94 °C—4 min
	ITS2R	5′-GCG GGT AAT CAC GTC TGA GCC GAG G-3′	(35 cycles) 94 °C—1 min, 60 °C—30 s and 72 °C—2 min; 72 °C—10 min
COI [23]	JB3	5′-GTCGTAACAAGGTTTCCGTA-3′	95 °C—5 min
	JB4	5′-GTCGTAACAAGGTTTCCGTA-3′	(35 cycles) 95 °C—30 s, 60 °C—1 min and 72 °C—1 min; 72 °C—5 min

**Table 2:** Geographic coordinates of the sampling locations around Lake Alqueva and the numbers of *Physella acuta* specimens collected during the August 2023 field survey for the Alqueva Project.

Sampling Location	Locality	Snails (n)	Latitude	Longitude
1	Parque de Merendas do Campinho	12	38.35418	−7443
2	Amieira (Caravanas)	1	38.29499	−7558
3	Praia Fluvial da Amieira	54	38.28977	−756
4	Amieira (Campo)	129	38.28972	−7566
5	Estrela (Albufeira)	30	38.26944	−7395
6	Praia dos Pescadores (Mourão)	10	38.40222	−7368
7	Praia Fluvial de Mourão	61	38.36806	−7354
8	Ancoradouro (Mourão)	173	38.37027	−7356
9	Herdade dos Delgados	10	38.38359	−7359
10	Mourão (Associação)	109	38.40555	−7348
11	Amareleja (Estrada)	54	38.34222	−7285
12	Herdade das Pipas	158	38.40428	−7423
13	Praia Fluvial Monsaraz—A	120	38.43519	−735
14	Praia Fluvial Monsaraz—B	112	38.43519	−735
15	Ancoradouro (Monsaraz)	64	38.42723	−7383
16	Praia Fluvial Azenhas d’El Rei	37	38.5473	−7303
17	Camping Rosário	75	38.6013	−7346
18	Juromenha	95	38.74666	−7235
Total		1304		

**Table 3:** GenBank accession numbers of the sequences obtained in this study.

	COI	ITS1	ITS2
Amieira			
*Al-C5*			PV013367
Mourão			
*Al-C8*	PV230524		PV013368
*Al-C12*		PV283215	
*Al-C13*			PV013369

## Data Availability

The data not presented in the article will be made available by the authors on request.

## Supplementary Materials

The following supporting information can be downloaded at https://www.mdpi.com/article/10.3390/pathogens14040304/s1, Figure S1: Phylogenetic tree based on COI sequences; Figure S2: Phylogenetic tree based on ITS1 sequence; Figure S3: NeighborNet network produced by SplitsTree6; Figure S4: Cercaria released from *Physella acuta*, visualized under 40× optical microscope, molecularly identified as *Posthodiplostomum* sp.
