# Supplementary material for: Physella acuta Confirmed as Intermediate Host of Posthodiplostomum sp. from Lake Alqueva, Portugal
Source: Pathogens. 2025 Mar 23;14(4):304. doi: 10.3390/pathogens14040304 (PMC12030160; doi:10.3390/pathogens14040304)
Supplement: Supplementary file 1 [file pathogens-14-00304-s001.zip › Suplemmentary information.pdf]

Supplementary information:

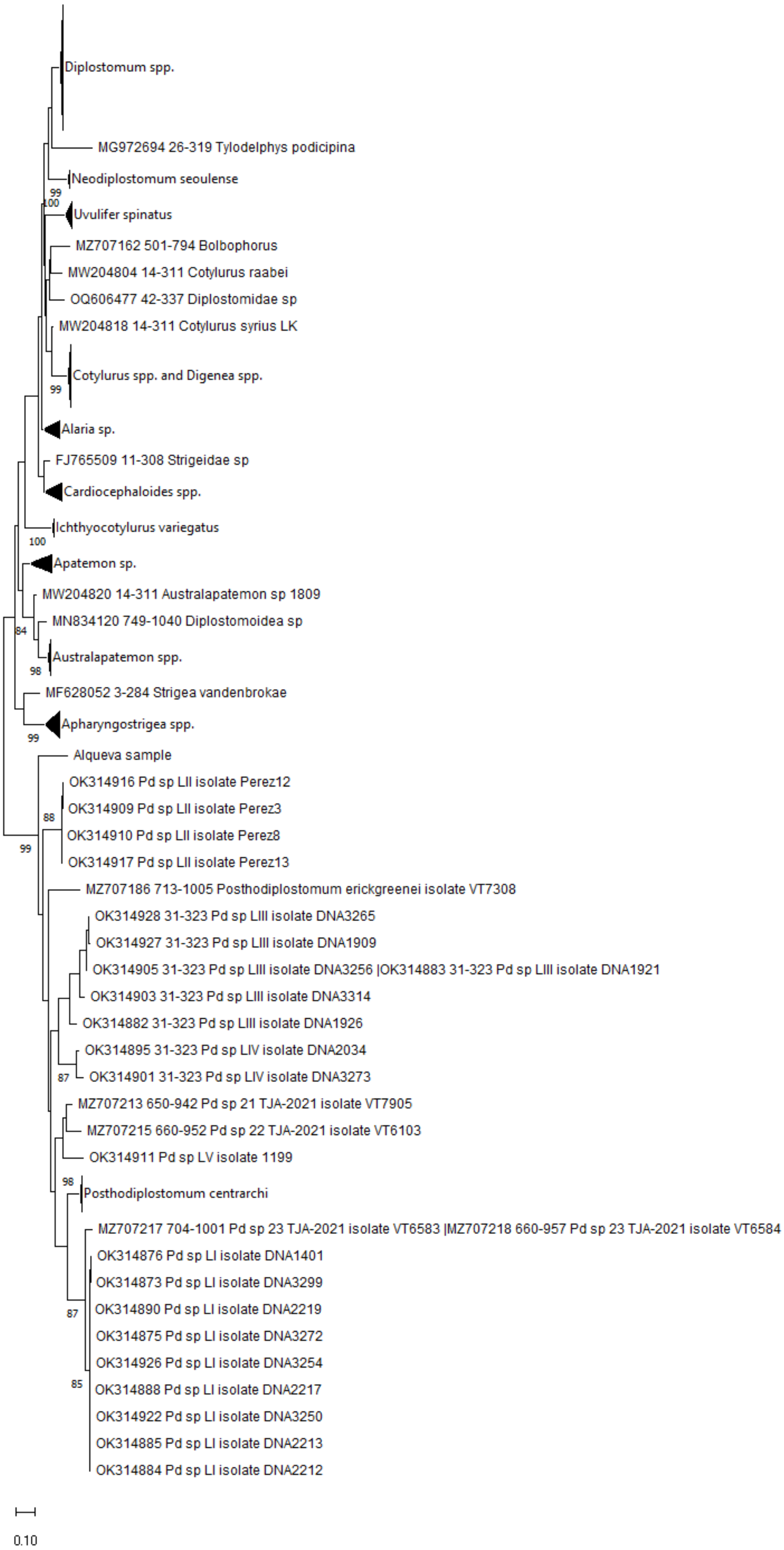

Figure S1: Phylogenetic Tree based on COI sequences. The Alqueva sample's COI sequence clusters with *Postodiplostomum* sp. with high bootstrap support, although the data suggests that it could belong to a novel lineage in this group. Maximum Likelihood tree produced in MEGA 11, using a General Time Reversible model (Nei M. & Kumar S. Molecular Evolution and Phylogenetics. 2000. Oxford University Press, New York) with a discrete Gamma distribution (5 categories; +G, parameter = 0,2151) from the dataset with a total of 269 positions after alignment and removal of gaps with uncertain alignment. The tree topology had the highest log likelihood (-4432,84) from an heuristic search conducted from initial trees obtained using Neighbor-Joining and BioNJ algorithms to a matrix of pairwise distances estimated using the Maximum Composite Likelihood (MCL) approach. Values above 80% for 100 bootstrap replicates are shown. Groups of species were grouped for improved visualization.

```
                   10        20        30        40        50        60
           ....|....|....|....|....|....|....|....|....|....|....|....|
1.         ATATGCATGGTTTTTATGAAAAATGATTCGGTTTTCAGTTATTCCGGGTTARTCGGTGCT
2.         ATTTGTATGGTTTACATGAATAATGATTCAGTGTTCAGTTATTCTGGGTTGGTAGGTGCA
3.         ATTTGTATGGTTTACATGAATAATGATTCAGTGTTCAGTTATTCTGGGTTGGTGGGTGCA
4.         ATTTGTATGGTTTATATGAATAATGATTCAGTGTTTAGTTATTCGGGGTTGGTAGGTGCA
5.         ATTTGTATGATTTATATGAAAAATGATTCTGTATTTAGTTATTCAGGTTTAGTAGGTGCT
6.         ATCTGTATGGTTTATATGAACAATGATTCTGTTTTTAGATATTCAGGTTTAGTGGGTGCC
7.         ATTTGTATGATTTATATGAAAAATGATTCTGTATTTAGTTATTCAGGTTTAGTAGGTGCT
8.         ATTTGTATGATTTATATGAAAAATGATTCTGTATTTAGTTATTCAGGTTTAGTAGGTGCT
9.         ATTTGTATGATTTATATGAAAAATGATTCTGTATTTAGTTATTCAGGTTTAGTAGGTGCT
10.        ATTTGTATGGTTTATATGAATAATGATTCTATATTTAGCTATTCTGGCTTGGTCGGTGCT
11.        ATATGTATGGTTTACATGAAAAATGATTCTGTTTTTAGATATTCTGGTTTAGTGGGTGCT
12.        ATTTGTATGGTGTATATGAAAAATGATTCTGTGTTTAGTTATTCTGGCTTAGTGGGTGCT
13.        ATTTGTATGGTGTATATGAAAAATGATTCTGTGTTTAGTTATTCTGGCCTAGTGGGTGCT
14.        ATTTGTATGGTGTATATGAAAAATGATTCTGTGTTTAGTTATTCTGGCTTAGTGGGTGCT
15.        ATTTGTATGGTGTATATGAAAAATGATTCTGTGTTTAGTTATTCTGGCTTAGTGGGTGCT
16.        ATTTGTATGGTTTATATGAATAATGATTCTATATTTAGTTATTCTGGTTTGGTTGGTGCT
17.        ATTTGTATGGTTTATATGAATAACGATTCAGTGTTCAGTTATTCGGGATTGGTAGGTGCA
18.        ATATGTATGGTGTATATGAAAAATGATTCTGTTTTTAGGTATACTGGTTTAGTTGGTGCA
19.        ATATGTATGGTGTATATGAAAAATGATTCTGTTTTTAGGTATACTGGTTTAGTCGGTGCA
20.        ATATGTATGGTGTATATGAAAAATGATTCTGTTTTTAGGTATACTGGTTTAGTTGGTGCA
21.        ATATGTATGATTTACATGAATAATGATTCAGTATTTAGTTATTCTGGTTTGGTGGGTGCC
22.        ATATGTATGGTGTATATGAAAAATGATTCTGTTTTTAGGTATACGGGTTTAGTTGGTGCA
23.        ATATGTATGGTGTATATGAAAAATGATTCTGTTTTTAGGTATACTGGTTTAGTTGGTGCA
24.        ATATGTATGGTGTATATGAAAAATGATTCTGTTTTTAGGTATACTGGTTTAGTTGGTGCA
25.        ATATGTATGGTGTATATGAAAAATGATTCTGTTTTTAGGTATACTGGTTTAGTTGGTGCA
26.        ATATGTATGGTGTACATGAAAAATGATTCTGTTTTCAGGTATACTGGTTTAGTTGGTGCA
27.        ATATGTATGGTGTATATGAAAAATGATTCTGTTTTTAGGTATACTGGTTTAGTTGGTGCA
28.        ATTTGTATGGTATACATGAAAAATGATTCTATTTTTAGTTATTCTGGTTTAGTTGGAGCA
29.        ATATGTATGGTGTATATGAAAAATGATTCTGTTTTTAGGTATACTGGTTTAGTTGGTGCA
30.        ATATGCTTGGTTTTTATGAATAAAGATTCTATTTTTAGTTATACTGGTTTAGTGGGTGCT
31.        ATATGTATGGTTTATATGAATAATGATTCAATATTTAGATATTCAGGGTTAGTAGGTGCT
32.        ATATGTTTGGTTTTTATGAATAAAGATTCTATTTTTAGTTATACAGGGTTAGTGGGTGCT
33.        ATATGTTTGGTTTTTATGAATAAAGATTCTATTTTTAGTTATACTGGGTTAGTAGGTGCT
34.        ATATGTTTGGTTTTTATGAATAAAGATTCTATTTTTAGTTATACTGGGTTAGTAGGTGCT
35.        ATATGTTTGGTATTTACTAATAATGATTCTGTTTTTAGTTATATGGGTTTAGTAGGTGCA
36.        ATATGTTTGGTATTTACTAATAATGATTCTGTTTTTAGTTATATGGGTTTAGTAGGTGCA
37.        ATATGCTTGGTTTTTATGAATAAAGATTCTATTTTTAGTTATACTGGTTTAGTGGGTGCT
38.        ATATGTTTAGTTTTTACTAAAAAAGATTCGGTTTTTAGTTATATGGGATTGGTGGGTGCT
```

```
39.   ATATGTTTAGTGTTTACTAATAAAGATTCTGTTTTTAGTTATATGGGTTTGGTAGGTGCT
40.   ATTTGTTTGGTTTTTACTAATAATGACTCTGTTTTTAGTTATATGGGTTTAGTTGGTGCT
41.   ATATGTTTAATTTTTACTAATAAAGATTCGGTTTTTAGTTATATGGGTTTAGTTGGTGCT
42.   ATTTGTTTGATTTTTACTAATAAAGATTCAGTATTTAGATATATGGGTTTAGTCGGGGCT
43.   ATATGTTTGGTTTTTACTAATAAAGATTCTGTTTTTAGTTATATGGGTTTAGTCGGTGCT
44.   ATTTGTTTAGTTTTTACTAATAAAGATTCGGTTTTTAGTTATTTAGGTTTAGTAGGTGCT
45.   ATTTGTTTGTTGTTTACTAATAAAGATTCTGTATTTAGGTATATGGGGTTGGTGGGTGCT
46.   ATTTGTTTGTTGTTTACTAATAAAGATTCTGTATTTAGGTATATGGGGTTGGTTGGTGCT
47.   ATTTGTTTGATTTTTTCTAATAATGATTCTGTTTTTAGTTATCTTGGTTTGGTGGGTGCT
48.   ATATGTTTGGTTTTTACTAATAAAGATTCTATTTTTAGTTATACTGGTTTGGTCGGTGCT
49.   ATATGTTTGGTTTTTACTAATAAAGATTCTATTTTTAGTTATACTGGTTTGGTCGGTGCT
50.   ATATGTTTAGTTTTTATGAATAAAGATTCTATTTTTAGTTATACTGGTTTAGTGGGTGCC
51.   ATTTGTTTAGTTTTTATGAATAAAGATTCTATTTTTAGTTATACTGGGTTGGTGGGAGCT
52.   ATTTGTTTGATTTTCACTAATAATGATTCTGTATTTAGTTATATGGGTTTAGTTGGTGCT
53.   ATTTGTTTGGTTTTTACTAATAATGATTCTGTTTTTAGTTATATGGGGTTGGTTGGGGCT
54.   ATTTGTTTGATTTTTTCTAATAATGATTCTGTTTTTAGTTATCTTGGTTTAGTGGGTGCT
55.   ATTTGTTTGATTTTTACTAATAAAGATTCAGTATTTAGATATATGGGTTTAGTTGGGGCT
56.   ATATGTTTGGTTTTTTCTAAAAATGATTCGGTTTTTAGTTATTTAGGCTTGGTAGGTGCT
57.   ATTTGTTTGATTTTTTCTAATAATGATTCTGTTTTTAGTTATCTTGGTTTGGTGGGTGCT
58.   ATATGTTTGGTTTTTACTAATAAAGATTCTATATTTAGTTATACTGGTTTGGTAGGTGCT
59.   ATATGTTTGGTTTTTACTAATAAAGATTCTATTTTTAGTTATACTGGTTTGGTTGGTGCT
60.   ATTTGTTTAGTTTTTACTAATAAAGATTCAGTTTTTAGTTATATGGGTTTAGTCGGTGCT
61.   ATTTGTTTGATTTTCACTAATAATGATTCTGTATTTAGTTATATGGGTTTAGTAGGTGCT
62.   ATTTGTTTGATTTTCACTAATAATGATTCTGTATTTAGTTATATGGGTTTAGTTGGTGCT
63.   ATATGTTTAGTATTTACTAATAATGATTCTGTTTTTAGTTATATGGGTCTAGTGGGTGCT
64.   ATTTGTTTAATTTTTACTAACAATGATTCTGTTTTTAGATATATGGGTTTGGTCGGTGCT
65.   ATTTGTTTAGTTTTTACTAATAAAGATTCTGTATTTAGTTATGTGGGTTTAGTTGGTGCT
66.   ATATGTTTAGTTTTTACTAATAAAGATTCTGTTTTTAGTTATTTAGGATTGGTAGGTGCC
67.   ATTTGTTTAACATTTACTAACAAAGATTCTGTTTTTAGTTATTTGGGTTTAGTGGGTGCA
68.   ATTTGTTTAACTTTTACTAATAAAGATTCTGTTTTTAGATATTTAGGTTTAGTCGGTGCT
69.   ATATGTTTAGTTTTCACTAATAAAGATTCTATTTTTAGTTATACTGGTTTAGTAGGTGCT
70.   ATTTGTTTAACTTTTACTAATAAAGATTCTGTTTTTAGATATTTAGGTTTAGTCGGTGCT
71.   ATATGCTTGGTTTTTATGAATAAAGATTCGATTTTTAGTTATACTGGTTTGGTAGGTGCT
72.   ATTTGTTTGATTTTCACTAATAATGATTCTGTATTTAGTTATATGGGTTTAGTAGGTGCT
73.   ATTTGTTTGATTTTCACTAATAATGATTCTGTATTTAGTTATATGGGTTTAGTTGGTGCT
74.   ATTTGTTTGATTTTCACTAATAATGATTCTGTATTTAGTTATATGGGTTTAGTAGGTGCT
75.   ATTTGTTTGATTTTCACTAATAATGATTCTGTATTTAGTTATATGGGTTTAGTTGGTGCT
76.   ATTTGTTTAACTTTTACTAATAAAGATTCTGTTTTTAGATATTTAGGTTTAGTCGGTGCT
77.   ATTTGTTTAACTTTTACTAATAAAGATTCTGTTTTTAGTTATTTGGGTTTAGTTGGTGCT
78.   ATATGTTTGGTTTTTACTAATAAAGATTCAATTTTTAGTTATACTGGTTTGGTAGGTGCT
79.   ATTTGTTTAACTTTTACTAATAAAGATTCTGTTTTTAGATATTTAGGTTTAGTCGGTGCT
80.   ATTTGTCTTGTTTTTACTAATAAAGATTCTGTTTTTAGTTATATGGGTTTAGTGGGTGCT

                     70        80        90       100       110       120
      ....|....|....|....|....|....|....|....|....|....|....|....|
1.    ATGTTTTCYATTGTTGTACYAGGTTGCATTGTATGAGTACATCATATGTTTATGGTGGGT
2.    ATGTTTTCTATAGTTGTTTTGGGTTGTATTGTCTGAGTACATCATATGTTTATGGTTGGT
3.    ATGTTTTCTATAGTTGTTTTAGGTTGTATTGTCTGAGTACATCATATGTTTATGGTTGGT
4.    ATGTTTTCTATAGTTGTTTTGGGTTGTATTGTCTGAGTACATCATATGTTTATGGTTGGT
5.    ATGTTTTCAATTGTTGTGCTAGGATGCATAGTTTGGGTGCATCATATGTTTATGGTTGGT
6.    ATGTTTTCTATAGTGGTGTTAGGTTGCATTGTGTGGGTTCATCATATGTTTATGGTGGGT
7.    ATGTTTTCAATTGTTGTGTTAGGATGCATAGTTTGGGTGCATCATATGTTTATGGTTGGT
8.    ATGTTTTCAATTGTTGTGCTAGGATGCATAGTTTGGGTGCATCATATGTTTATGGTTGGT
9.    ATGTTTTCAATTGTTGTGTTAGGATGCATAGTTTGGGTGCATCATATGTTTATGGTTGGT
10.   ATGTTTTCAATAGTTGTTCTAGGTTGTATAGTTTGAGTACATCATATGTTTATGGTGGGG
11.   ATGTTTTCTATAGTTGTGTTAGGTTGTATTGTGTGAGTTCATCACATGTTTATGGTGGGT
12.   ATGTTTTCTATAGTGGTTTTAGGGTGTATCGTTTGGGTGCACCATATGTTTATGGTGGGA
13.   ATGTTTTCTATAGTGGTTTTAGGGTGTATTGTTTGGGTGCACCATATGTTTATGGTGGGA
14.   ATGTTTTCTATAGTGGTTTTAGGGTGTATCGTTTGGGTGCACCATATGTTTATGGTGGGA
15.   ATGTTTTCTATAGTGGTTTTAGGGTGTATTGTTTGGGTGCACCATATGTTTATGGTGGGA
16.   ATGTTTTCGATAGTTGTTCTTGGTTGTATAGTATGAGTGCATCATATGTTTATGGTAGGG
```

17. ATGTTTTCAATAGTTGTTTTGGGTTGTATTGTTTGGGTGCATCATATGTTTATGGTTGGT
18. ATGTTTTCTATAGTGGTGTTAGGGTGTATAGTTTGAGTTCATCATATGTTTATGGTTGGA
19. ATGTTTTCTATAGTGGTGTTAGGGTGTATAGTTTGAGTTCATCATATGTTTATGGTTGGA
20. ATGTTTTCTATAGTGGTGTTAGGGTGTATAGTTTGAGTTCATCATATGTTTATGGTTGGA
21. ATGTTTTCAATAGTTGTTTTGGGTTGTATTGTTTGGGTTCACCACATGTTTATGGTTGGT
22. ATGTTTTCTATAGTGGTGTTAGGGTGTATAGTTTGAGTTCATCATATGTTTATGGTTGGA
23. ATGTTTTCTATAGTGGTGTTAGGGTGTATAGTTTGAGTTCATCATATGTTTATGGTTGGA
24. ATGTTTTCTATAGTGGTGTTAGGGTGTATAGTTTGAGTTCATCATATGTTTATGGTTGGG
25. ATGTTTTCTATAGTGGTGTTAGGGTGTATAGTTTGAGTTCATCATATGTTTATGGTTGGG
26. ATGTTTTCTATAGTGGTTTTGGGTTGTATAGTTTGGGTTCATCATATGTTTATGGTTGGA
27. ATGTTTTCTATAGTGGTGTTAGGGTGTATAGTTTGAGTTCATCATATGTTTATGGTTGGA
28. ATGTTTTCTATAGTTGTATTAGGGTGTATTGTGTGGGTGCATCATATGTTTATGGTTGGT
29. ATGTTTTCTATAGTGGTGTTAGGGTGTATAGTTTGAGTTCATCATATGTTTATGGTTGGA
30. ATGTTTTCAATTGTTGTTTTAGGTTGTATTGTTTGGGTTCATCATATGTTCATGGTTGGT
31. ATGTTCTCCATAGTGGTGTTAGGATGTATTGTGTGGGTTCATCACATGTTCATGGTAGGT
32. ATGTTTTCTATTGTTGTTTTAGGTTGTATTGTTTGAGTTCATCATATGTTTATGGTTGGT
33. ATGTTTTCTATTGTTGTTTTAGGTTGTATTGTTTGAGTTCATCATATGTTTATGGTTGGT
34. ATGTTTTCTATTGTTGTTTTAGGTTGTATTGTTTGAGTTCATCATATGTTTATGGTTGGT
35. ATGTTTTCTATTGTTGTTTTAGGGTGTATAGTTTGAGCTCATCATATGTTTATGGTTGGT
36. ATGTTTTCTATTGTTGTTTTAGGGTGTATAGTTTGAGCTCATCATATGTTTATGGTTGGT
37. ATGTTTTCAATTGTTGTTTTAGGTTGTATCGTTTGGGTTCATCATATGTTCATGGTTGGT
38. ATGTTTTCTATTGTTGTTTTAGGTTGTATTGTGTGGGTGCATCATATGTTTATGGTTGGT
39. ATGTTTTCTATTGTTGTTTTAGGTTGTATAGTTTGAGTTCATCATATGTTTATGGTTGGT
40. ATGTTTTCTATTGTTGTTTTAGGTTTTATTGTTTGGGTGCATCATATGTTTATGGTCGGT
41. ATGTTTTCTATTGTGGTTTTGGGTTGTATAGTTTGAGTTCATCATATGTTTATGGTTGGT
42. ATGTTTTCTATAGTTGTTTTAGGTTTCATTGTTTGGGTACATCATATGTTTATGGTTGGT
43. ATGTTTTCTATTGTTGTTTTAGGTTGTATAGTTTGGGTTCATCATATGTTTATGGTGGGT
44. ATGTTTTCAATTGTGATTTTAGGTTGTATTGTTTGGGTTCATCATATGTTTATGGTTGGT
45. ATGTTTTCTATTGTTGTTTTAGGTTGTATAGTTTGAGTTCATCATATGTTTATGGTGGGT
46. ATGTTTTCTATTGTTGTTTTAGGTTGTATAGTTTGAGTTCATCATATGTTTATGGTGGGT
47. ATGTTTGCGATAGTTGTTTTAGGTTGTATTGTTTGGGTGCATCATATGTTTATGGTGGGT
48. ATGTTTTCTATAGTTGTTTTAGGTTGTATTGTTTGGGTTCATCATATGTTTATGGTTGGT
49. ATGTTTTCTATAGTTGTTTTAGGTTGTATTGTTTGGGTTCATCATATGTTTATGGTTGGT
50. ATGTTTTCAATTGTTGTTTTAGGTTGTATTGTTTGAGTTCATCACATGTTCATGGTTGGT
51. ATGTTTTCTATTGTTGTTTTGGGTTGTATTGTTTGGGTGCATCATATGTTTATGGTTGGT
52. ATGTTTTCTATAGTTGTTTTAGGTTTTATTGTATGAGTCCATCATATGTTTATGGTAGGT
53. ATGTTTTCTATTGTTGTTTTAGGGTGTATTGTTTGAGTTCATCATATGTTTATGGTTGGT
54. ATGTTTGCTATAGTTGTTTTAGGTTGCATTGTTTGGGTTCATCATATGTTTATGGTAGGA
55. ATGTTTTCTATAGTTGTTTTAGGTTTCATTGTTTGGGTACATCATATGTTTATGGTTGGT
56. ATGTTTTCTATAGTTGTATTAGGGTGCATAGTTTGGGTTCATCATATGTTTATGGTTGGT
57. ATGTTTGCGATAGTTGTTTTAGGTTGTATTGTTTGGGTGCATCATATGTTTATGGTGGGT
58. ATGTTTTCTATAGTTGTTTTAGGTTGTATTGTTTGGGTTCATCATATGTTTATGGTTGGT
59. ATGTTTTCTATAGTTGTTTTAGGTTGTATTGTTTGGGTTCATCATATGTTTATGGTTGGT
60. ATGTTTTCTATAGTTGTTTTGGGTTGTATAGTTTGGGTTCATCATATGTTTATGGTGGGT
61. ATGTTTTCTATAGTTGTTTTAGGGTTTATTGTATGAGTTCATCATATGTTTATGGTAGGT
62. ATGTTTTCTATAGTTGTTTTAGGTTTTATTGTATGAGTCCATCATATGTTTATGGTAGGT
63. ATGTTTTCTATTGTTGTTTTAGGGTGTATAGTTTGAGCTCATCATATGTTTATGGTTGGT
64. ATGTTTTCTATTGTTGTTTTAGGTTTTATTGTATGGGTTCATCACATGTTTATGGTGGGT
65. ATGTTCTCTATAGTGGTTTTAGGTTGTATAGTTTGGGTACATCATATGTTTATGGTTGGT
66. ATGTTTGCTATTGTGGTGTTAGGTTGTATAGTTTGAGTTCATCATATGTTTATGGTGGGG
67. ATGTTTTCTATTGTTGTGTTAGGTTGTATAGTTTGAGTGCATCATATGTTTATGGTAGGT
68. ATGTTTTCTATAGTTGTATTAGGTTGTATAGTGTGAGTACATCATATGTTTATGGTAGGG
69. ATGTTTTCAATTGTTGTTCTAGGTTGTATTGTGTGAGTTCATCATATGTTTATGGTTGGT
70. ATGTTTTCTATAGTTGTGTTAGGTTGTATAGTGTGAGTACATCATATGTTTATGGTAGGG
71. ATGTTTTCAATTGTTGCTTTGGGTTGTGTTGTTTGAGTTCATCATATGTTTATGATTGGT
72. ATGTTTTCTATAGTTGTTTTAGGGTTTATTGTATGAGTTCATCATATGTTTATGGTAGGT
73. ATGTTTTCTATAGTTGTTTTAGGTTTTATTGTATGAGTCCATCATATGTTTATGGTAGGT
74. ATGTTTTCTATAGTTGTTTTAGGGTTTATTGTATGAGTTCATCATATGTTTATGGTAGGT
75. ATGTTTTCTATAGTTGTTTTAGGTTTTATTGTATGAGTCCATCATATGTTTATGGTAGGT
76. ATGTTTTCTATAGTTGTATTAGGTTGTATAGTGTGAGTACATCATATGTTTATGGTAGGG
77. ATGTTTTCTATAGTTGTGTTAGGTTGTATAGTTTGGGTACATCATATGTTTATGGTAGGT

```
78.   ATGTTTTCAATTGTAGTTTTAGGTTGTATAGTTTGAGTCCATCATATGTTTATGGTTGGT
79.   ATGTTTTCTATAGTTGTGTTAGGTTGTATAGTGTGAGTACATCATATGTTTATGGTAGGG
80.   ATGTTTTCTATTGTGGTTTTAGGTTGTATAGTTTGGGTTCATCATATGTTTATGGTGGGT

                130       140       150       160       170       180
      ....|....|....|....|....|....|....|....|....|....|....|....|
1.    TTAGAGYTTCGAAGGTTGATTTTTTTTAGYTCTMCAMCYMTGGTTATAGGGATTCCCACR
2.    TTGGAGTTTCGAAGGTTGATATTTTTCAGTTCTACTACTATGGTTATTGGGATTCCCACG
3.    TTGGAGTTTCGAAGGTTGATATTTTTCAGTTCTACTACTATGGTTATTGGGATTCCTACA
4.    TTGGAGTTTCGAAGGTTGATATTTTTCAGTTCCACTACTATGGTTATCGGGATTCCCACA
5.    TTAGAACTTCGAAGGTTAATTTTTTTTAGTTCTACTACTATGGTTATTGGAATTCCAACT
6.    TTAGAGTTTCGAAGGTTAGTCTTTTTTAGATCTACCACTATGGTCATTGGTATTCCGACA
7.    TTAGAACTTCGAAGGTTAATTTTTTTTAGTTCTACTACTATGGTTATTGGAATTCCAACT
8.    TTAGAACTTCGAAGGTTAATTTTTTTTAGTTCTACTACAATGGTTATTGGTATTCCAACT
9.    TTAGAACTTCGAAGGTTAATTTTTTTTAGTTCTACTACAATGGTTATTGGTATTCCAACT
10.   TTGGAGTTTCGAAGTTTGATTTTTTTTAGTTCTACTACAATGGTCATTGGCATACCTACA
11.   TTAGAATTTCGAAGGTTGGTTTTCTTCAGATCTACTACTATGGTTATTGGTATTCCTACT
12.   TTAGAGTTACGTAGGTTAATTTTTTTTAGTTCAACTACAATGGTTATAGGTATACCTACG
13.   TTAGAGTTACGTAGGTTAATTTTTTTTAGTTCAACTACAATGGTTATAGGTATACCTACG
14.   TTAGAGTTACGTAGGTTAATTTTTTTTAGTTCAACTACAATGGTTATAGGTATACCAACG
15.   TTAGAGTTACGTAGGTTAATTTTTTTTAGTTCAACTACAATGGTTATAGGTATACCTACG
16.   TTGGAGTTTCGAAGTTTGATTTTTTTTAGCTCTACTACAATGGTTATTGGTATACCTACA
17.   TTAGAGTTTCGAAGGCTAATATTTTTTAGTTCCACTACTATGGTTATTGGTATTCCCACC
18.   TTAGAATTGCGTAGTTTAATATTTTTTAGTTCAACTACAATGGTAATAGGAATTCCTACG
19.   TTAGAATTGCGTAGTTTAATATTTTTTAGTTCAACTACAATGGTAATAGGAATTCCTACG
20.   TTAGAATTGCGTAGTTTAATATTTTTTAGTTCAACCACAATGGTAATAGGAATTCCTACG
21.   TTAGAGTTTCGAAGGTTGATATTTTTTAGTTCCACTACAATGGTTATTGGGATTCCTACC
22.   TTAGAATTGCGTAGTTTAATATTTTTTAGTTCAACTACAATGGTAATAGGAATTCCTACG
23.   TTAGAATTGCGTAGTTTAATATTTTTTAGTTCAACTACAATGGTAATAGGAATTCCTACA
24.   TTAGAATTACGTAGTTTAATATTTTTTAGTTCAACTACAATGGTAATAGGAATTCCTACG
25.   TTAGAATTGCGTAGTTTAATATTTTTTAGTTCAACTACAATGGTAATAGGAATTCCTACG
26.   TTAGAATTACGGAGTTTAATATTTTTTAGTTCAACTACAATGGTTATCGGTATTCCCACG
27.   TTAGAATTGCGTAGTTTAATATTTTTTAGTTCAACTACAATGGTAATAGGAATTCCTACT
28.   TTAGAATTTCGAAGATTAGTTTTTTTTAGTTCGACAACTATGGTTATTGGTATACCTACT
29.   TTGGAATTGCGTAGTTTAATATTTTTTAGTTCAACTACAATGGTAATAGGAATTCCTACG
30.   TTAGAGTTTCGTAGTTTAGTCTTTTTTAGGTCTACAACAATGGTTATCGGTATACCAACT
31.   CTGGAGTTTCGAAGTTTAATTTTTTTTAGGTCAACCACCATGGTTATTGGTATACCTACA
32.   TTAGAATTTCGTAGTTTAGTCTTTTTTAGATCTACAACTATGGTTATCGGCATACCTACT
33.   TTAGAATTTCGTAGTTTAGTTTTTTTTAGATCTACAACTATGGTTATCGGCATACCTACC
34.   TTAGAATTTCGTAGTTTAGTTTTTTTTAGATCTACAACTATGGTTATCGGCATACCTACT
35.   TTAGAGTTTCGTAGACTTGTTTTTTTTAGTTCTACTACTATGGTTATAGGGATACCAACT
36.   TTAGAGTTTCGTAGACTTGTTTTTTTTAGTTCTACTACTATGGTTATAGGGATACCAACT
37.   TTAGAGTTTCGTAGTTTAGTCTTTTTTAGGTCTACAACAATGGTTATCGGTATACCAACT
38.   TTAGAATTTCGTAGACTTGTTTTTTTTAGTTCTACTACTATGGTGATAGGTATACCCACA
39.   TTGGAGTTTCGTAGGCTTATATTTTTTAGTTCTACTACAATGGTTATAGGTATTCCAACC
40.   TTAGAGTTTCGTAGTCTTGTTTTTTTTAGTTCTACTACTATGGTTATTGGTATACCAACA
41.   TTAGAGTTTCGTAGTTTGGTTTTTTTTAGTTCTACTACTATGGTTATTGGTATTCCCACA
42.   TTGGAGTTTCGCAGTCTTGTGTTTTTTAGTTCCACAACAATGGTGATTGGTATTCCAACG
43.   TTGGAGTTTCGTAGTCTTGTGTTTTTTAGTTCTACTACGATGGTTATAGGTATTCCGACA
44.   GTTGAGTTTCGTAGTCTTGTTTTTTTTAGTTCGACCACTATGGTTATAGGTATTCCTACG
45.   TTAGAGTTTCGCAGTTTGGTTTTTTTTAGCTCTACCACTATGGTTATAGGTATACCAACC
46.   TTAGAGTTTCGCAGTTTGGTTTTTTTTAGCTCTACCACTATGGTTATAGGTATACCAACC
47.   ATAGAGTTTCGTAGTTTGATTTTTTTTAGTTCAACTACAATGATTATTGGTATTCCAACG
48.   TTAGAATTTCGTAGTCTTGTTTTTTTTAGTTCTACAACTATGGTTATTGGTATACCGACA
49.   TTAGAATTTCGTAGTCTTGTTTTTTTTAGTTCTACAACTATGGTTATTGGTATACCGACG
50.   TTAGAGTTTCGTAGTTTAGTCTTTTTTAGTTCTACTACAATGGTTATCGGTATACCAACA
51.   TTAGAATTTCGTAGTTTAGTTTTTTTTAGTTCAACCACTATGGTTATAGGTATACCAACA
52.   TTAGAATTTCGTAGTCTTGTTTTTTTTAGTTCTACTACTATGGTTATTGGTATTCCCACA
53.   TTAGAGTTTCGTAGTCTTGTTTTTTTTAGTTCTACAACAATGGTTATTGGTATACCCACA
54.   ATAGAGTTTCGTAGTTTAATTTTTTTTAGTTCTACTACAATGATTATAGGTATTCCTACG
55.   TTGGAGTTTCGCAGTCTTGTGTTTTTTAGTTCCACAACAATGGTGATTGGTATTCCAACG
```

```
56.   ATAGAGTTCCGTAGTCTTGTGTTTTTTAGCTCTACTACTATGGTTATAGGTATTCCGACG
57.   ATAGAGTTTCGTAGTTTAATTTTTTTTAGTTCAACTACAATGATTATTGGTATTCCAACG
58.   TTAGAATTTCGTAGTCTTGTTTTTTTTAGTTCTACAACTATGGTTATTGGTATACCGACG
59.   TTAGAATTTCGTAGTCTTGTTTTTTTTAGTTCTACAACTATGGTTATTGGTATACCGACG
60.   TTGGAGTTTCGTAGTCTTATATTTTTTAGTTCTACTACTATGGTAATTGGTATTCCAACA
61.   TTAGAGTTTCGTAGTCTTGTTTTTTTTAGTTCTACTACTATGGTTATTGGTATTCCTACA
62.   TTAGAATTTCGTAGTCTTGTTTTTTTTAGTTCTACTACTATGGTTATTGGTATTCCTACA
63.   TTAGAATTTCGTAGTCTTGTTTTTTTTAGTTCTACCACTATGGTTATTGGTATACCAACT
64.   TTAGAGTTTCGTAGTCTTGTTTTTTTTAGTTCTACAACTATGGTGATTGGAATACCAACG
65.   TTAGAGTTTCGTAGTTTGGTTTTTTTTAGGTCTGTTACTATGGTGATTGGTATTCCGACG
66.   GTAGAGTTTCGTAGTCTTGTTTTTTTTAGATCTACTACTATGATTATAGGTGTGCCAACC
67.   GTCGAGTTTCGAAGTCTTGTCTTTTTTAGGTCTACTACTATGGTTATTGGTATTCCCACA
68.   GTTGAGTTTCGGAGTCTTGTTTTTTTTAGTTCAACTACTATGGTTATTGGTATTCCCACT
69.   TTAGAGTTCCGTAGACTTGTTTTTTTTAGTTCTACAACTATGGTTATCGGAATACCAACT
70.   GTTGAGTTTCGAAGCCTTGTTTTTTTTAGTTCAACTACTATGGTTATTGGTATTCCCACC
71.   TTAGAAATGCGCAGTCTTGTTTTTTTTAGCTCTACAACTATGGTTATTGGTATACCAACG
72.   TTAGAGTTTCGTAGTCTTGTTTTTTTTAGTTCTACTACTATGGTTATTGGTATTCCTACA
73.   TTAGAATTTCGTAGTCTTGTTTTTTTTAGTTCTACTACTATGGTTATTGGTATTCCTACA
74.   TTAGAGTTTCGTAGTCTTGTTTTTTTTAGTTCTACTACTATGGTTATTGGTATTCCTACA
75.   TTAGAATTTCGTAGTCTTGTTTTTTTTAGTTCTACTACTATGGTTATTGGTATTCCTACA
76.   GTTGAGTTTCGGAGTCTTGTTTTTTTTAGTTCAACTACTATGGTTATTGGTATTCCCACC
77.   GTTGAGTTTCGAAGTCTTGTTTTTTTTAGTTCGACTACTATGGTTATAGGTGTTCCCACG
78.   TTAGAGTTTCGCAGTCTTGTTTTTTTTAGCTCTACCACTATGGTTATCGGAATACCAACA
79.   GTTGAGTTTCGGAGTCTTGTTTTTTTTAGTTCAACTACTATGGTTATTGGTATTCCCACA
80.   TTAGAATTTCGTAGTCTTGTATTTTTTAGTTCTACTACTATGGTTATTGGTATTCCTACC

                190       200       210       220       230       240
      ....|....|....|....|....|....|....|....|....|....|....|....|
1.    GGGATAAAGGTGTTTTCCYGGTTAATAATGCTGCGYAGATCTTGATATCGCTTTTCASAT
2.    GGTATAAAGGTTTTTTCCTGGTTAATAATGTTGCGTAGTTCTTGATATCGGTTAAGGGAT
3.    GGTATAAAGGTTTTTTCCTGGTTAATAATGTTGCGTAGTTCTTGATACCGGTTAAGGGAT
4.    GGTATAAAGGTTTTTTCCTGGTTAATAATGTTGCGTAGTTCTTGATATCGGTTAAGGGAT
5.    GGTATAAAGGTATTTTCTTGGTTGATAATGTTGCGGAGTTCCTGATATCGTTTTACTGAT
6.    GGTATTAAGGTTTTTTCTTGGTTAATAATGTTGCGAAGTTCTTGATATCGGTTAGGTGAT
7.    GGTATAAAGGTATTTTCTTGGTTGATAATGTTGCGGAGTTCCTGATATCGTTTTACTGAT
8.    GGTATAAAGGTATTTTCTTGATTGATAATGTTGCGGAGTTCCTGATATCGTTTTACTGAT
9.    GGTATAAAGGTATTTTCTTGGTTGATAATGTTGCGGAGTTCCTGATATCGTTTTACTGAT
10.   GGAATAAAGGTTTTTTCCTGATTGATAATGTTGCGAAGATCTTGATACCGTTTAAGAGAT
11.   GGTATAAAGGTTTTTTCTTGATTAATAATGTTGCGGAGTTCTTGATATCGACTAAGTGAT
12.   GGAATAAAGGTTTTTTCCTGATTAATAATGTTGCGCAGGTCGTGGTATCGTTTTAGTGAT
13.   GGAATAAAGGTTTTTTCCTGATTAATAATGTTGCGCAGGTCGTGGTATCGTTTTAGTGAT
14.   GGAATAAAGGTTTTTTCCTGATTAATAATGTTGCGCAGGTCGTGGTATCGTTTTAGTGAT
15.   GGAATAAAGGTTTTTTCCTGATTAATAATGTTGCGCAGGTCGTGGTATCGTTTTAGTGAT
16.   GGAATAAAGGTTTTTTCCTGATTGATAATGTTGCGAAGATCTTGATATCGTTTAGGGGAT
17.   GGTATTAAGGTTTTTTCCTGATTAATAATGTTGCGTAGTTCTTGATATCGGTTGGGGGAT
18.   GGTATAAAGGTTTTTTCGTGGTTAATAATGTTGCGAAGTTCTTGATATCGTTTTAGTGAT
19.   GGTATAAAGGTTTTTTCGTGGTTAATAATGTTGCGAAGTTCTTGATATCGTTTTAGTGAT
20.   GGTATAAAGGTTTTTTCGTGGTTAATAATGTTGCGAAGTTCTTGATATCGTTTTAGTGAT
21.   GGAATAAAGGTTTTTTCTTGATTAATAATGTTGCGTAGTTCTTGATATCGGTTAGGGGAT
22.   GGTATAAAGGTTTTTTCGTGGTTAATAATGTTGCGAAGTTCTTGATATCGTTTTAGTGAT
23.   GGTATAAAGGTTTTTTCGTGGTTAATAATGTTGCGAAGTTCTTGATATCGTTTTAGTGAT
24.   GGTATAAAGGTTTTTTCGTGGTTAATAATGTTGCGAAGTTCTTGATATCGTTTTAGTGAT
25.   GGTATAAAGGTTTTTTCGTGGTTAATAATGTTGCGAAGTTCTTGATATCGTTTTAGTGAT
26.   GGTATCAAGGTTTTTTCGTGGTTGATAATGTTGCGAAGTTCTTGATATCGTTTTAGTGAT
27.   GGTATAAAGGTTTTTTCGTGGTTAATAATGTTGCGAAGTTCTTGATATCGTTTTAGTGAT
28.   GGTATAAAGGTTTTTTCTTGGTTGATAATGTTGCGTAGTTCTTGGTATCGTATGGGGGAT
29.   GGTATAAAGGTTTTTTCGTGGTTAATAATGTTGCGAAGTTCTTGATATCGTTTTAGTGAT
30.   GGTATTAAGGTTTTTTCTTGGTTATATATGTTGCGTGGTGCGTGGTATCGTATGAGTGAT
31.   GGTATAAAGGTTTTTTCTTGATTAATAATGTTACGAAGTTCTTGATATCGTTTAGGGGAT
32.   GGGATAAAGGTTTTTTCTTGATTGTATATGTTGCGTGGTGCTTGGTATCGTATGAGTGAT
33.   GGGATAAAGGTTTTTTCTTGATTGTATATGTTGCGTGGTGCTTGGTATCGTATGAGTGAT
```

```
34.    GGGATAAAGGTTTTTTCTTGATTGTATATGTTGCGTGGTGCTTGGTATCGTATGAGTGAT
35.    GGAATAAAGGTTTTTTCTTGGATATATATGTTGCGTAGTTCTTGATATCATATTATGAGT
36.    GGAATAAAGGTTTTTTCTTGGATATATATGTTGCGTAGTTCTTGATATCATATTATGAGT
37.    GGTATTAAGGTTTTTTCTTGGTTATATATGTTGCGTGGTGCGTGGTATCGTATGAGTGAT
38.    GGTATAAAGGTTTTTTCTTGAGTTTATATGTTACGTGGTAGTTGGTTTCGTATGATGGAT
39.    GGAATTAAGGTTTTTTCTTGAGTTTATATGTTGCGTGGTTCATGGTTTCGTGGTACGGAT
40.    GGTATTAAGGTTTTTTCTTGAATATATATGTTGCGTGGCTCTTGGTTTCGTATAATGGAT
41.    GGTATTAAGGTTTTTTCCTGGATTTATATGTTGCGTGGTGCTTGGTTTCGTATTTTGGAT
42.    GGGATTAAGGTTTTTTCCTGGGTTTATATGTTGCGTAGTTCTTGATTTCGATTTAGTGAT
43.    GGTATTAAGGTTTTTTCTTGAATTTATATGTTGCGTGGGTCGTGGTTTCGTGGTTTAGAT
44.    GGTATAAAGGTTTTTTCTTGATTGTATATGTTACGTAGTTCTTGATTTCGTTTAAAAGAT
45.    GGTATTAAGGTTTTTTCTTGAATATATATGTTGCGCGGTGCTTGATTTCGCGTTTATGAT
46.    GGTATTAAGGTTTTTTCTTGAATATATATGTTGCGCGGTGCTTGATTTCGCGTTTATGAT
47.    GGTATAAAGGTTTTTTCTTGGCTTTATATGCTGCGTAGTTCTTGGTTTCGTGTGTCGGAT
48.    GGTATTAAGGTTTTTTCATGATTATATATGTTGCGCGGAGCTTGGTATCGTATGAATGAT
49.    GGTATTAAGGTTTTTTCATGATTATATATGTTGCGCGGAGCTTGGTATCGTATGAATGAT
50.    GGTATTAAGGTTTTTTCATGGTTATACATGTTGCGAGGGGCGTGGTACCGTATGAGAGAT
51.    GGTATTAAGGTTTTTTCTTGATTATATATGTTGCGCGGTGCTTGGTATCGTATGAACGAT
52.    GGTATTAAGGTTTTTTCATGAATTTATATGTTACGTGGATCTTGATTTCGTATTATGGAT
53.    GGTATTAAGGTTTTTTCTTGAATTTATATGTTGCGTAGTGGTTGGTTTCGTTTAATGGAT
54.    GGGATTAAGGTTTTTTCTTGGCTTTATATGTTGCGAAGCTCTTGGTTTCGTGTATCGGAT
55.    GGGATTAAGGTTTTTTCCTGGGTTTATATGTTGCGTAGTTCTTGATTTCGATTTAGTGAT
56.    GGGATTAAGGTTTTTTCTTGGATTTATATGTTGCGGAGTTCTTGATTTCGGTTGTCGGAT
57.    GGTATAAAGGTTTTTTCTTGGCTTTATATGCTGCGTAGTTCTTGGTTTCGTGTGTCGGAT
58.    GGTATTAAGGTTTTTTCATGATTATATATGTTGCGCGGAGCTTGATATCGTATGAATGAT
59.    GGTATTAAGGTTTTTTCATGATTATATATGTTGCGCGGAGCTTGGTATCGTATGAATGAT
60.    GGGATTAAGGTTTTTTCTTGGATTTATATGTTGCGTGGGTCTTGGTTTCGTGGTATGGAC
61.    GGTATTAAGGTTTTTTCATGAATTTATATGTTACGTGGATCTTGATTTCGTATTATGGAT
62.    GGTATTAAGGTTTTTTCATGAATTTATATGTTACGTGGATCTTGATTTCGTATTATGGAT
63.    GGAATAAAGGTTTTTTCTTGAATATATATGTTGCGTAGTTCTTGATATCATATTATGAGT
64.    GGTATTAAGGTTTTTTCTTGAATTTATATGTTGCGGGGTTGTTGATTTCGTATAATGGAT
65.    GGTATTAAGGTTTTTTCTTGATTATATATGCTGCGGACATCTTGGTATCGTATGGGTGAT
66.    GGGATAAAGGTTTTTTCTTGATTATATATGTTGCGTAGTTCTTGGTTTCGTTTAAATGAT
67.    GGTATTAAGGTTTTTTCTTGGCTTTATATGTTGCGTAGTTCTTGATTCCGCTTATCTGAT
68.    GGTATAAAGGTTTTTTCTTGACTTTATATGTTGCGGGGTTCTTGATTTCGTTTATCGGAT
69.    GGTATTAAGGTCTTTTCGTGATTATATATGTTACGTGGTTCTTGATACCGTATGAATGAG
70.    GGTATAAAGGTTTTTTCTTGACTTTATATGTTGCGGGGTTCTTGATTTCGTTTATCGGAT
71.    GGTATTAAGGTTTTTTCTTGATTATATATGTTGCGTGGTGCTTGGTACCGTATGGATGAT
72.    GGTATTAAGGTTTTTTCGTGAATTTATATGTTACGTGGATCTTGGTTTCGTATTATGGAT
73.    GGTATTAAGGTTTTTTCGTGAATTTATATGTTACGTGGATCTTGGTTTCGTATTATGGAT
74.    GGTATTAAGGTTTTTTCGTGAATTTATATGTTACGTGGATCTTGGTTTCGTATTGTGGAT
75.    GGTATTAAGGTTTTTTCATGAATTTATATGTTACGTGGGTCTTGATTTCGTATTATGGAT
76.    GGTATAAAGGTTTTTTCTTGACTTTATATGTTGCGGGGTTCTTGATTTCGTTTATCGGAT
77.    GGTATTAAGGTTTTTTCTTGGCTTTATATGTTGCGTGGTTCTTGATTCCGTTTATCTGAT
78.    GGGATTAAGGTTTTTTCATGACTTTATATGTTACGGGGTTCTTGGTATCGTATGGGTGAT
79.    GGTATAAAGGTTTTTTCTTGACTTTATATGTTGCGTGGTTCTTGATTTCGTTTATCGGAT
80.    GGTATTAAGGTTTTTTCTTGGATTTATATGTTGCGTGGTGCTTGATTTCGTATCTTGGAT

                    250       260
           ....|....|....|....|....|....
1.            CCAGTATTGTGGTGAATTATAGGGTTTAT
2.            CCTGTGTTGTGGTGAATTATTGGATTTAT
3.            CCTGTGTTGTGGTGAATTATTGGATTTAT
4.            CCTGTGTTGTGGTGGATTATTGGATTTAT
5.            CCCGTATTATGATGAATTGTTGGGTTTAT
6.            CCCGTACTGTGGTGAATAATTGGTTTTAT
7.            CCCGTATTATGATGAATTGTTGGGTTTAT
8.            CCCGTATTATGATGAATTGTTGGGTTTAT
9.            CCCGTATTATGATGAATTGTTGGGTTTAT
10.           CCGGTACTTTGGTGAATTATTGGTTTTAT
11.           CCTGTTCTGTGGTGAATCATTGGTTTTAT
```

12. CCCGTACTGTGGTGAATAGTGGGGTTTAT
13. CCCGTACTGTGGTGAATAGTGGGGTTTAT
14. CCCGTACTGTGGTGAATAGTGGGGTTTAT
15. CCCGTACTGTGGTGAATAGTGGGGTTTAT
16. CCGGTGCTTTGATGAATTATTGGTTTTAT
17. CCTGTGTTGTGGTGGATTATTGGATTTAT
18. CCAGTACTTTGGTGAATTGTTGGTTTTAT
19. CCAGTACTTTGGTGAATTGTTGGTTTTAT
20. CCAGTACTTTGGTGAATTGTTGGTTTTAT
21. CCGGTGCTATGGTGAATTATTGGGTTTAT
22. CCAGTACTTTGGTGAATTGTTGGTTTTAT
23. CCAGTACTTTGGTGAATTGTTGGTTTTAT
24. CCAGTACTTTGGTGAATTGTTGGTTTTAT
25. CCAGTACTTTGGTGAATTGTTGGTTTTAT
26. CCAGTACTTTGGTGAATAATTGGTTTTAT
27. CCAGTACTTTGGTGAATTGTTGGTTTTAT
28. CCTGTTTTATGATGAATTATAGGTTTTAT
29. CCAGTACTTTGGTGAATTGTTGGTTTTAT
30. CCTATATTTTGGTGAATTTTAGGTTTTAT
31. CCCGTTCTCTGATGAATAATTGGTTTTAT
32. CCTATATTTTGGTGAATCTTGGGTTTTAT
33. CCTATATTTTGGTGAATCTTGGGTTTTAT
34. CCTATATTTTGGTGAATCTTGGGTTTTAT
35. CCTATTTTTTGGTGAATTATGGGTTTCAT
36. CCTATTTTTTGGTGAATTATGGGTTTTAT
37. CCTATATTTTGGTGAATTTTAGGTTTTAT
38. CCAATATTTTGGTGAATTTTAGGTTTTAT
39. CCGGTATTTTGGTGAATATTGGGCTTTAT
40. CCTGTTTTTTGGTGAATTTTAGGGTTTAT
41. CCAATTCTTTGGTGGATTTTAGGTTTTAT
42. CCAGTTTTTTGGTGGATTATCGGTTTTAT
43. CCTGTGTTTTGGTGAATTTTGGGTTTTAT
44. CCTGTTTTTTGGTGGATAATTGGTTTTAT
45. CCCATATTTTGGTGAATTTTGGGTTTTAT
46. CCCATATTTTGGTGAATTTTGGGTTTTAT
47. CCTGTTTTTTGGTGAGTTGTTGGATTTAT
48. CCTATATTTTGATGAATATTAGGTTTTAT
49. CCTATATTTTGATGAATATTAGGTTTTAT
50. CCTATATTTTGGTGAATACTAGGTTTTAT
51. CCTATATTTTGGTGAATTTTAGGGTTTAT
52. CCTGTTTTTTGATGGATTTTAGGATTTAT
53. CCTATTTTTTGATGGGTTATAGGTTTTAT
54. CCCGTTTTTTGATGGGTTGTCGGGTTTAT
55. CCAGTTTTTTGGTGGATTATCGGTTTTAT
56. CCTGTTTATTGGTGAGTAGTTGGTTTTAT
57. CCTGTTTTTTGGTGAGTTGTTGGATTTAT
58. CCTATATTCTGATGAATATTAGGTTTTAT
59. CCTATATTTTGATGAATATTAGGTTTTAT
60. CCTGTATTTTGGTGAATTTTGGGTTTTAT
61. CCTGTTTTTTGATGGATTTTAGGATTTAT
62. CCTGTTTTTTGATGGATTTTAGGATTTAT
63. CCTGTTTTTTGGTGAATAATGGGTTTTAT
64. CCTATATTCTGGTGAATTTTAGGTTTTAT
65. CCAATATTTTGATGGATTTTGGGGTTTAT
66. CCTGTTTTTTGATGAATTATTGGTTTTAT
67. CCCGTTTTTTGGTGAGTAATCGGTTTTAT
68. CCAGTTTTTTGGTGGGTAATTGGTTTTAT
69. CCTATATTTTGGTGGATATTAGGATTTAT
70. CCAGTTTTTTGGTGGGTAATTGGTTTTAT
71. CCCATTTTTTGATGAATTTTAGGTTTTAT
72. CCTGTTTTTTGATGGATTTTAGGATTTAT

73. CCTGTTTTTTGATGGATTTTAGGATTTAT
74. CCTGTTTTTTGATGGATTTTAGGATTTAT
75. CCTGTTTTTTGATGGATTTTAGGATTTAT
76. CCAGTTTTTTGGTGGGTAATTGGTTTTAT
77. CCTGTTTTTTGGTGAATCATTGGTTTCAT
78. CCTATATTTTGGTGAATATTAGGATTTAT
79. CCAGTTTTTTGGTGGGTAATCGGTTTTAT
80. CCTATATTGTGATGAATTATCGGTTTTAT

Full sequence titles:

1. Alqueva sample
2. OK314928_31-323_Pd_sp_LIII_isolate_DNA3265_
3. OK314927_31-323_Pd_sp_LIII_isolate_DNA1909_
4. OK314905_31-323_Pd_sp_LIII_isolate_DNA3256_|OK314883_31-323_Pd_sp_LIII_isolate_DNA1921_
5. OK314916__Pd_sp_LII_isolate_Perez12_
6. MZ707213_650-942_Pd_sp_21_TJA-2021_isolate_VT7905_
7. OK314909__Pd_sp_LII_isolate_Perez3_
8. OK314917__Pd_sp_LII_isolate_Perez13_
9. OK314910__Pd_sp_LII_isolate_Perez8_
10. OK314895_31-323_Pd_sp_LIV_isolate_DNA2034_
11. MZ707215_660-952_Pd_sp_22_TJA-2021_isolate_VT6103
12. MH536512_7689-7986_Posthodiplostomum_centrarchi
13. MZ707178_643-940_Posthodiplostomum_centrarchi_isolate_VT6579_
14. MZ707175_662-959_Posthodiplostomum_centrarchi_isolate_VT6581_
15. MZ707179_659-947_Posthodiplostomum_centrarchi_isolate_VT6574_
16. OK314901_31-323_Pd_sp_LIV_isolate_DNA3273_
17. OK314903_31-323_Pd_sp_LIII_isolate_DNA3314_
18. OK314888__Pd_sp_LI_isolate_DNA2217_
19. OK314885__Pd_sp_LI_isolate_DNA2213_
20. OK314884__Pd_sp_LI_isolate_DNA2212_
21. OK314882_31-323_Pd_sp_LIII_isolate_DNA1926_
22. OK314926__Pd_sp_LI_isolate_DNA3254_
23. OK314890__Pd_sp_LI_isolate_DNA2219_
24. OK314876__Pd_sp_LI_isolate_DNA1401_
25. OK314873__Pd_sp_LI_isolate_DNA3299_
26. MZ707217_704-1001_Pd_sp_23_TJA-2021_isolate_VT6583_|MZ707218_660-957_Pd_sp_23_TJA-2021_isolate_VT6584_
27. OK314875__Pd_sp_LI_isolate_DNA3272_
28. MZ707186_713-1005_Posthodiplostomum_erickgreenei_isolate_VT7308_
29. OK314922__Pd_sp_LI_isolate_DNA3250_
30. OQ330724_680-977_Diplostomum_spathaceum_isolate_Bt_An_2009_
31. OK314911__Pd_sp_LV_isolate_1199_
32. MZ323260_635-932_Diplostomum_huronense_isolate_VT6419_
33. MZ323259_623-920_Diplostomum_huronense_isolate_VT7870_
34. MZ323258_617-914_Diplostomum_huronense_isolate_VT7869_
35. MF568677__Uvulifer_spinatus_
36. MF568676__Uvulifer_spinatus_
37. MZ323277_652-949_Diplostomum_spathaceum_
38. MZ707162_501-794_Bolbophorus_
39. MN817945_659-956_Cardiocephaloides_longicollis_
40. MW204818_14-311_Cotylurus_syrius_LK_
41. KT223036__Alaria_sp_AlaspWI-01_
42. AJ301898_11-301_Ichthyocotylurus_variegatus_
43. FJ765509_11-308_Strigeidae_sp_
44. OR030108_673-964_Apatemon_sp_
45. OP185214_51-342_Neodiplostomum_seoulense_
46. OP185212_51-342_Neodiplostomum_seoulense
47. OR030105_705-997_Apharyngostrigea_sp_
48. LC599713_2-299_Diplostomum_sp_A_

49. LC599712_2-299_Diplostomum_sp_
50. OQ330723_680-977_Diplostomum_spathaceum_
51. MZ323254_632-911_Diplostomum_gavium_
52. MZ558239_63-360_Digenea_sp_
53. OQ606477_42-337_Diplostomidae_sp_
54. NC_059570_8022-8313_Apharyngostrigea_pipientis_
55. AJ301896_11-301_Ichthyocotylurus_variegatus_
56. MF628052_3-284_Strigea_vandenbrokae_
57. OR030106_705-997_Apharyngostrigea_sp_isolate_PYO056.6_
58. LC599721_2-299_Diplostomum_sp_B_
59. LC599714_2-299_Diplostomum_sp_A_
60. MH536508_7844-8141_Cardiocephaloides_medioconiger_
61. MZ558237_63-360_Cotylurus_sp_
62. MZ558235_42-339_Cotylurus_sp_
63. MF568674__Uvulifer_spinatus_
64. MW204804_14-311_Cotylurus_raabei_
65. MG972694_26-319_Tylodelphys_podicipina_
66. JX051348_26-318_Aff._Apatemon_sp_1_
67. MN834120_749-1040_Diplostomoidea_sp_
68. OQ658623_2-281_Australapatemon_sp_ZP536_
69. OR044097_680-971_Diplostomum_numericum_
70. OP715848_14-293_Australapatemon_burti_voucher_50_
71. MZ323294_643-934_Diplostomum_sp_VVT3_isolate_VT7024_
72. MZ558238_42-339_Digenea_sp_dig28_
73. MZ558236_63-360_Cotylurus_sp_cot3
74. MZ558233_42-339_Cotylurus_sp_cot3_
75. MZ558227_63-360_Digenea_sp_dig26_
76. MW204829_14-293_Australapatemon_sp_PG5
77. MW204820_14-311_Australapatemon_sp_1809_
78. MZ323250_695-986_Diplostomum_alascense_isolate_VT7673_
79. MW204821_14-293_Australapatemon_sp_PS2_
80. MT328806_38-335_Alaria_sp_F237_

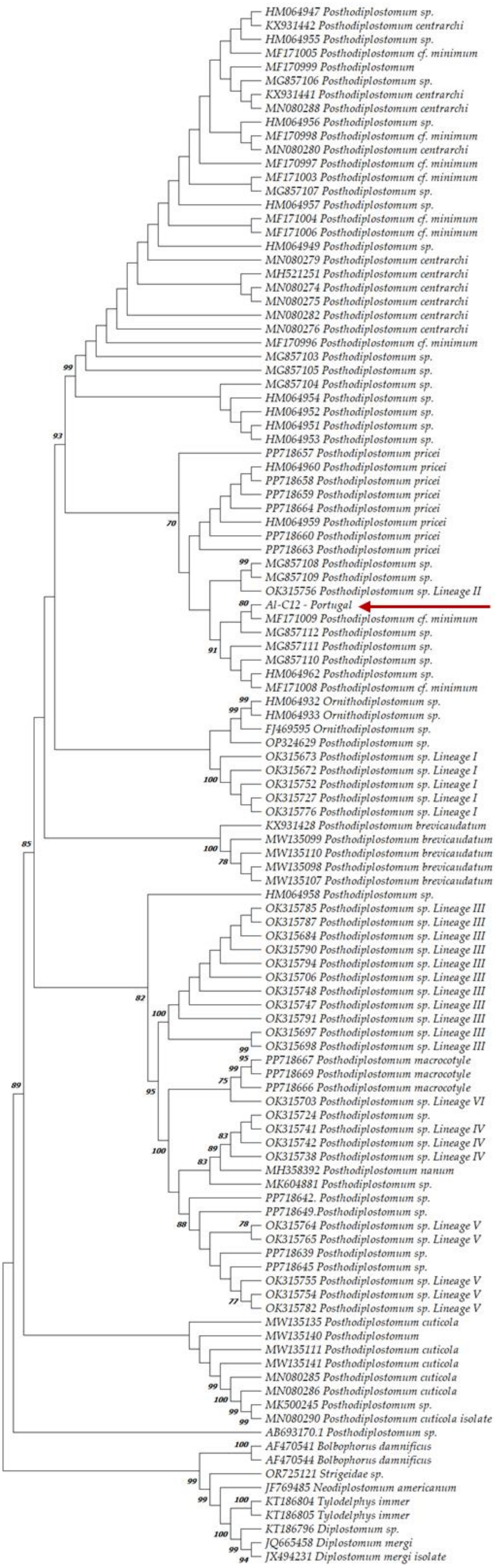
HM064947 Posthodiplostomum sp.
KX931442 Posthodiplostomum centrarchi
HM064955 Posthodiplostomum sp.
MF171005 Posthodiplostomum cf. minimum
MF170999 Posthodiplostomum
MG857106 Posthodiplostomum sp.
KX931441 Posthodiplostomum centrarchi
MN080288 Posthodiplostomum centrarchi
HM064956 Posthodiplostomum sp.
MF170998 Posthodiplostomum cf. minimum
MN080280 Posthodiplostomum centrarchi
MF170997 Posthodiplostomum cf. minimum
MF171003 Posthodiplostomum cf. minimum
MG857107 Posthodiplostomum sp.
HM064957 Posthodiplostomum sp.
MF171004 Posthodiplostomum cf. minimum
MF171006 Posthodiplostomum cf. minimum
HM064949 Posthodiplostomum sp.
MN080279 Posthodiplostomum centrarchi
MH521251 Posthodiplostomum centrarchi
MN080274 Posthodiplostomum centrarchi
MN080275 Posthodiplostomum centrarchi
MN080282 Posthodiplostomum centrarchi
MN080276 Posthodiplostomum centrarchi
MF170996 Posthodiplostomum cf. minimum
MG857103 Posthodiplostomum sp.
MG857105 Posthodiplostomum sp.
MG857104 Posthodiplostomum sp.
HM064954 Posthodiplostomum sp.
HM064952 Posthodiplostomum sp.
HM064951 Posthodiplostomum sp.
HM064953 Posthodiplostomum sp.
PP718657 Posthodiplostomum pricei
HM064960 Posthodiplostomum pricei
PP718658 Posthodiplostomum pricei
PP718659 Posthodiplostomum pricei
PP718664 Posthodiplostomum pricei
HM064959 Posthodiplostomum pricei
PP718660 Posthodiplostomum pricei
PP718663 Posthodiplostomum pricei
MG857108 Posthodiplostomum sp.
MG857109 Posthodiplostomum sp.
OK315756 Posthodiplostomum sp. Lineage II
Al-C12 - Portugal
MF171009 Posthodiplostomum cf. minimum
MG857112 Posthodiplostomum sp.
MG857111 Posthodiplostomum sp.
MG857110 Posthodiplostomum sp.
HM064962 Posthodiplostomum sp.
MF171008 Posthodiplostomum cf. minimum
HM064932 Ornithodiplostomum sp.
HM064933 Ornithodiplostomum sp.
FJ469595 Ornithodiplostomum sp.
OP324629 Posthodiplostomum sp.
OK315673 Posthodiplostomum sp. Lineage I
OK315672 Posthodiplostomum sp. Lineage I
OK315752 Posthodiplostomum sp. Lineage I
OK315727 Posthodiplostomum sp. Lineage I
OK315776 Posthodiplostomum sp. Lineage I
KX931428 Posthodiplostomum brevicaudatum
MW135099 Posthodiplostomum brevicaudatum
MW135110 Posthodiplostomum brevicaudatum
MW135098 Posthodiplostomum brevicaudatum
MW135107 Posthodiplostomum brevicaudatum
HM064958 Posthodiplostomum sp.
OK315785 Posthodiplostomum sp. Lineage III
OK315787 Posthodiplostomum sp. Lineage III
OK315684 Posthodiplostomum sp. Lineage III
OK315790 Posthodiplostomum sp. Lineage III
OK315794 Posthodiplostomum sp. Lineage III
OK315706 Posthodiplostomum sp. Lineage III
OK315748 Posthodiplostomum sp. Lineage III
OK315747 Posthodiplostomum sp. Lineage III
OK315791 Posthodiplostomum sp. Lineage III
OK315697 Posthodiplostomum sp. Lineage III
OK315698 Posthodiplostomum sp. Lineage III
PP718667 Posthodiplostomum macrocotyle
PP718669 Posthodiplostomum macrocotyle
PP718666 Posthodiplostomum macrocotyle
OK315703 Posthodiplostomum sp. Lineage VI
OK315724 Posthodiplostomum sp.
OK315741 Posthodiplostomum sp. Lineage IV
OK315742 Posthodiplostomum sp. Lineage IV
OK315738 Posthodiplostomum sp. Lineage IV
MH358392 Posthodiplostomum nanum
MK604881 Posthodiplostomum sp.
PP718642. Posthodiplostomum sp.
PP718649.Posthodiplostomum sp.
OK315764 Posthodiplostomum sp. Lineage V
OK315765 Posthodiplostomum sp. Lineage V
PP718639 Posthodiplostomum sp.
PP718645 Posthodiplostomum sp.
OK315755 Posthodiplostomum sp. Lineage V
OK315754 Posthodiplostomum sp. Lineage V
OK315782 Posthodiplostomum sp. Lineage V
MW135135 Posthodiplostomum cuticola
MW135140 Posthodiplostomum
MW135111 Posthodiplostomum cuticola
MW135141 Posthodiplostomum cuticola
MN080285 Posthodiplostomum cuticola
MN080286 Posthodiplostomum cuticola
MK500245 Posthodiplostomum sp.
MN080290 Posthodiplostomum cuticola isolate
AB693170.1 Posthodiplostomum sp.
AF470541 Bolbophorus damnificus
AF470544 Bolbophorus damnificus
OR725121 Strigeidae sp.
JF769485 Neodiplostomum americanum
KT186804 Tylodelphys immer
KT186805 Tylodelphys immer
KT186796 Diplostomum sp.
JQ665458 Diplostomum mergi
JX494231 Diplostomum mergi isolate

Supplementary figure S2: Phylogenetic tree based on ITS1 sequences. Neighbor-Joining tree produced in MEGA 11, using a Tamura 3 parameter model (Nei M. & Kumar S). Molecular Evolution and Phylogenetics. 2000. Oxford University Press, New York) with Gamma distribution. The Alqueva sample's ITS1 sequence clusters with *Postodiplostomum cf. minimum* with high bootstrap support.

1. Al-C12 - Portugal
2. AB693170_Posthodiplostomum sp.
3. HM064947_Posthodiplostomum sp.
4. HM064949_Posthodiplostomum sp.
5. HM064951_Posthodiplostomum sp.
6. HM064952_Posthodiplostomum sp.
7. HM064953_Posthodiplostomum sp.
8. HM064954_Posthodiplostomum sp.
9. HM064955_Posthodiplostomum sp.
10. HM064956_Posthodiplostomum sp.
11. HM064957_Posthodiplostomum sp.
12. HM064958_Posthodiplostomum sp.
13. HM064959_Posthodiplostomum pricei
14. HM064960_Posthodiplostomum pricei
15. HM064962_Posthodiplostomum sp.
16. KX931428_Posthodiplostomum brevicaudatum
17. KX931441_Posthodiplostomum centrarchi
18. KX931442_Posthodiplostomum centrarchi
19. MF170996_Posthodiplostomum cf. minimum
20. MF170997_Posthodiplostomum cf. minimum
21. MF170998_Posthodiplostomum cf. minimum
22. MF170999_Posthodiplostomum
23. MF171003_Posthodiplostomum cf. minimum
24. MF171004_Posthodiplostomum cf. minimum
25. MF171005_Posthodiplostomum cf. minimum
26. MF171006_Posthodiplostomum cf. minimum
27. MF171008_Posthodiplostomum cf. minimum
28. MF171009_Posthodiplostomum cf. minimum
29. MG857103_Posthodiplostomum sp.
30. MG857104_Posthodiplostomum sp.
31. MG857105_Posthodiplostomum sp.
32. MG857106_Posthodiplostomum sp.
33. MG857107_Posthodiplostomum sp.
34. MG857108_Posthodiplostomum sp.
35. MG857110_Posthodiplostomum sp.
36. MG857111_Posthodiplostomum sp.
37. MG857112_Posthodiplostomum sp.
38. MH358392_Posthodiplostomum nanum
39. MH521251_Posthodiplostomum centrarchi
40. MK500245_Posthodiplostomum sp.
41. MK604881_Posthodiplostomum sp.
42. MN080274_Posthodiplostomum centrarchi
43. MN080275_Posthodiplostomum centrarchi
44. MN080276_Posthodiplostomum centrarchi
45. MN080279_Posthodiplostomum centrarchi

46. MN080280_Posthodiplostomum centrarchi
47. MN080282_Posthodiplostomum centrarchi
48. MN080285_Posthodiplostomum cuticola
49. MN080286_Posthodiplostomum cuticola
50. MN080288_Posthodiplostomum centrarchi
51. MN080290_Posthodiplostomum cuticola isolate
52. MW135098_Posthodiplostomum brevicaudatum
53. MW135099_Posthodiplostomum brevicaudatum
54. MW135107_Posthodiplostomum brevicaudatum
55. MW135110_Posthodiplostomum brevicaudatum
56. MW135111_Posthodiplostomum cuticola
57. MW135135_Posthodiplostomum cuticola
58. MW135140_Posthodiplostomum
59. MW135141_Posthodiplostomum cuticola
60. OK315672_Posthodiplostomum sp. Lineage I
61. OK315673_Posthodiplostomum sp. Lineage I
62. OK315684_Posthodiplostomum sp. Lineage III
63. OK315697_Posthodiplostomum sp. Lineage III
64. OK315698_Posthodiplostomum sp. Lineage III
65. OK315703_Posthodiplostomum sp. Lineage VI
66. OK315706_Posthodiplostomum sp. Lineage III
67. OK315724_Posthodiplostomum sp.
68. OK315727_Posthodiplostomum sp. Lineage I
69. OK315738_Posthodiplostomum sp. Lineage IV
70. OK315741_Posthodiplostomum sp. Lineage IV
71. OK315742_Posthodiplostomum sp. Lineage IV
72. OK315747_Posthodiplostomum sp. Lineage III
73. OK315748_Posthodiplostomum sp. Lineage III
74. OK315752_Posthodiplostomum sp. Lineage I
75. OK315754_Posthodiplostomum sp. Lineage V
76. OK315755_Posthodiplostomum sp. Lineage V
77. OK315756_Posthodiplostomum sp. Lineage II
78. OK315764_Posthodiplostomum sp. Lineage V
79. OK315765_Posthodiplostomum sp. Lineage V
80. OK315776_Posthodiplostomum sp. Lineage I
81. OK315782_Posthodiplostomum sp. Lineage V
82. OK315785_Posthodiplostomum sp. Lineage III
83. OK315787_Posthodiplostomum sp. Lineage III
84. OK315790_Posthodiplostomum sp. Lineage III
85. OK315791_Posthodiplostomum sp. Lineage III
86. OK315794_Posthodiplostomum sp. Lineage III
87. OP324629_Posthodiplostomum sp.
88. PP718639_Posthodiplostomum sp.
89. PP718642_Posthodiplostomum sp.
90. PP718645_Posthodiplostomum sp.
91. PP718649_Posthodiplostomum sp.
92. PP718658_Posthodiplostomum pricei
93. PP718659_Posthodiplostomum pricei
94. PP718660_Posthodiplostomum pricei
95. PP718663_Posthodiplostomum pricei
96. PP718664_Posthodiplostomum pricei
97. PP718666_Posthodiplostomum macrocotyle
98. PP718667_Posthodiplostomum macrocotyle

99. PP718669_Posthodiplostomum macrocotyle
100. AF470541_Bolbophorus damnificus
101. AF470544_Bolbophorus damnificus
102. FJ469595_Ornithodiplostomum sp.
103. HM064932_Ornithodiplostomum sp.
104. HM064933_Ornithodiplostomum sp.
105. JF769485_Neodiplostomum americanum
106. JQ665458_Diplostomum mergi
107. JX494231_Diplostomum mergi isolate
108. KT186796_Diplostomum sp.
109. KT186804_Tylodelphys immer
110. KT186805_Tylodelphys immer
111. OR725121_Strigeidae sp.

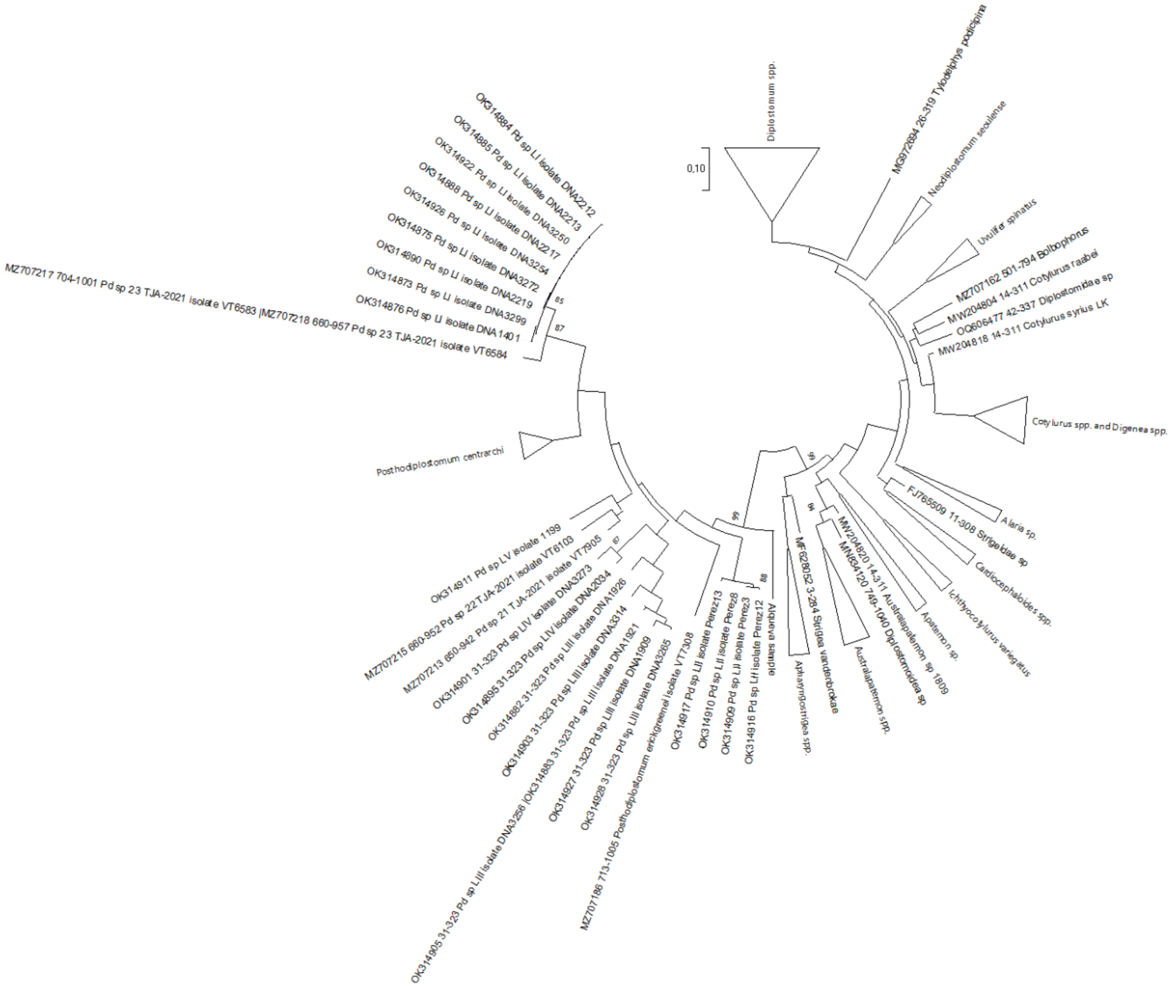

Supplementary figure S3: NeighborNet network produced by SplitsTree6. Alqueva's sample Al-C8 clustered with *Postodiplostomum* sp. isolates OK314916, OK314909, OK314910 and OK314917 with high homology.

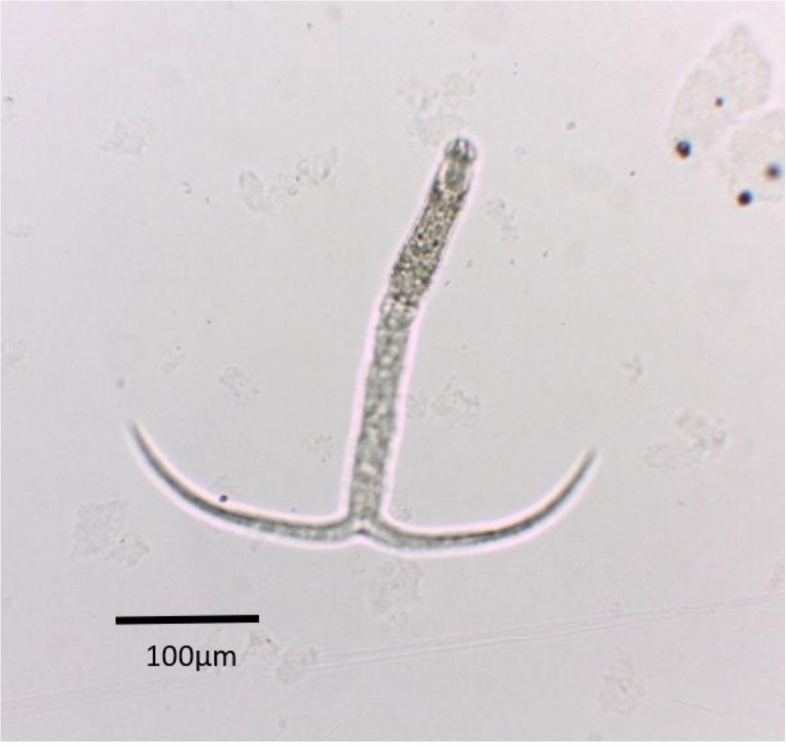

Supplementary figure S4: Cercaria released from *Physella acuta*, visualized under 40× optical microscope, molecularly identified as *Posthodiplostomum* sp..
