# Supplementary figures and images for: Physella acuta Confirmed as Intermediate Host of Posthodiplostomum sp. from Lake Alqueva, Portugal
Source: Pathogens. 2025 Mar 23;14(4):304. doi: 10.3390/pathogens14040304 (PMC12030160; doi:10.3390/pathogens14040304)

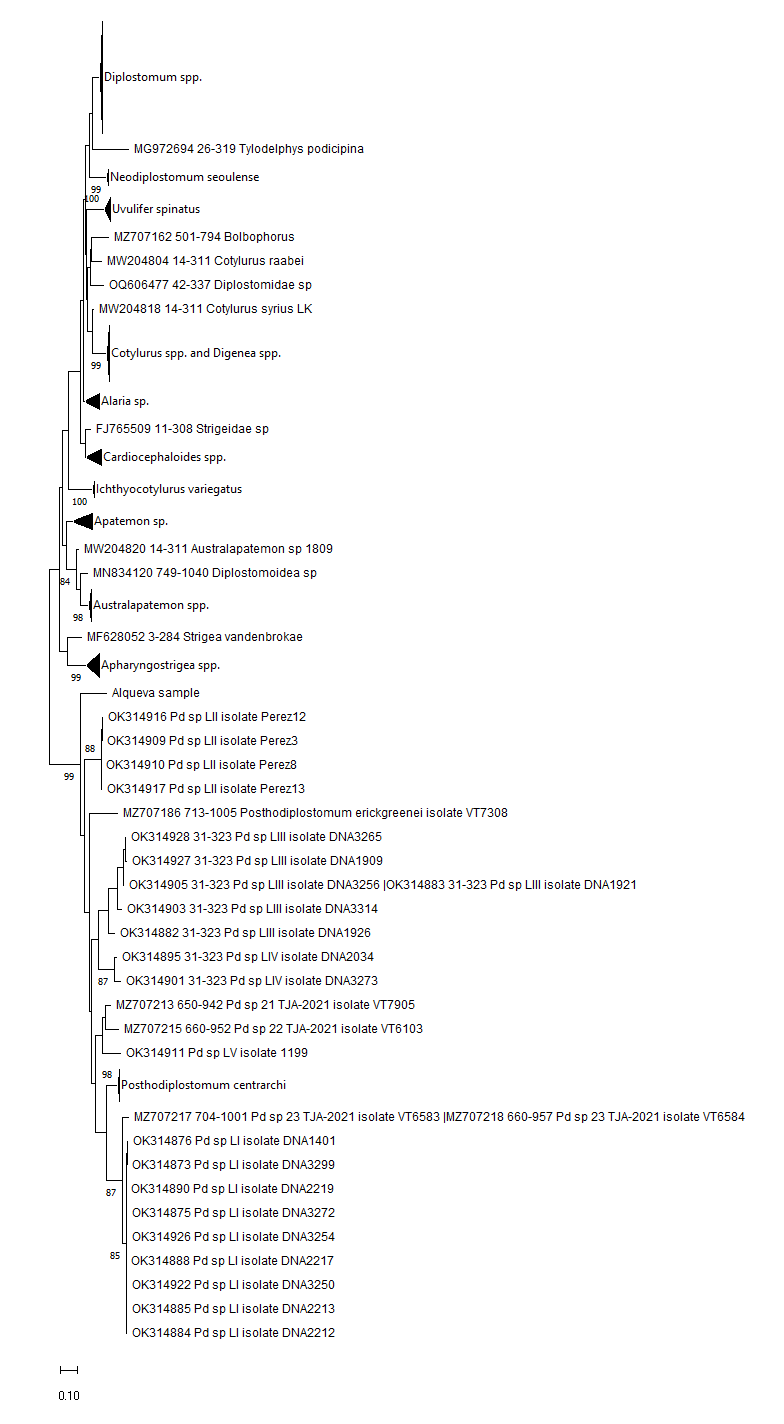

Supplement: Supplementary file 1 [file pathogens-14-00304-s001.zip › Supplementary figures/S1.tif]

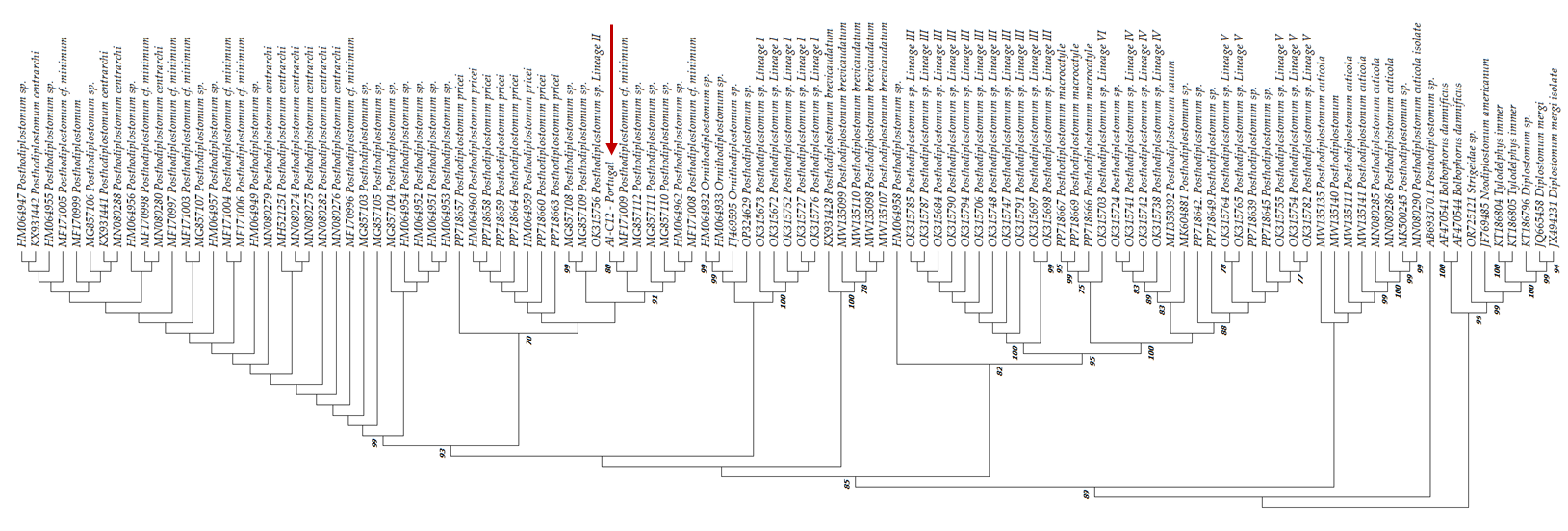

Supplement: Supplementary file 1 [file pathogens-14-00304-s001.zip › Supplementary figures/S2.png]

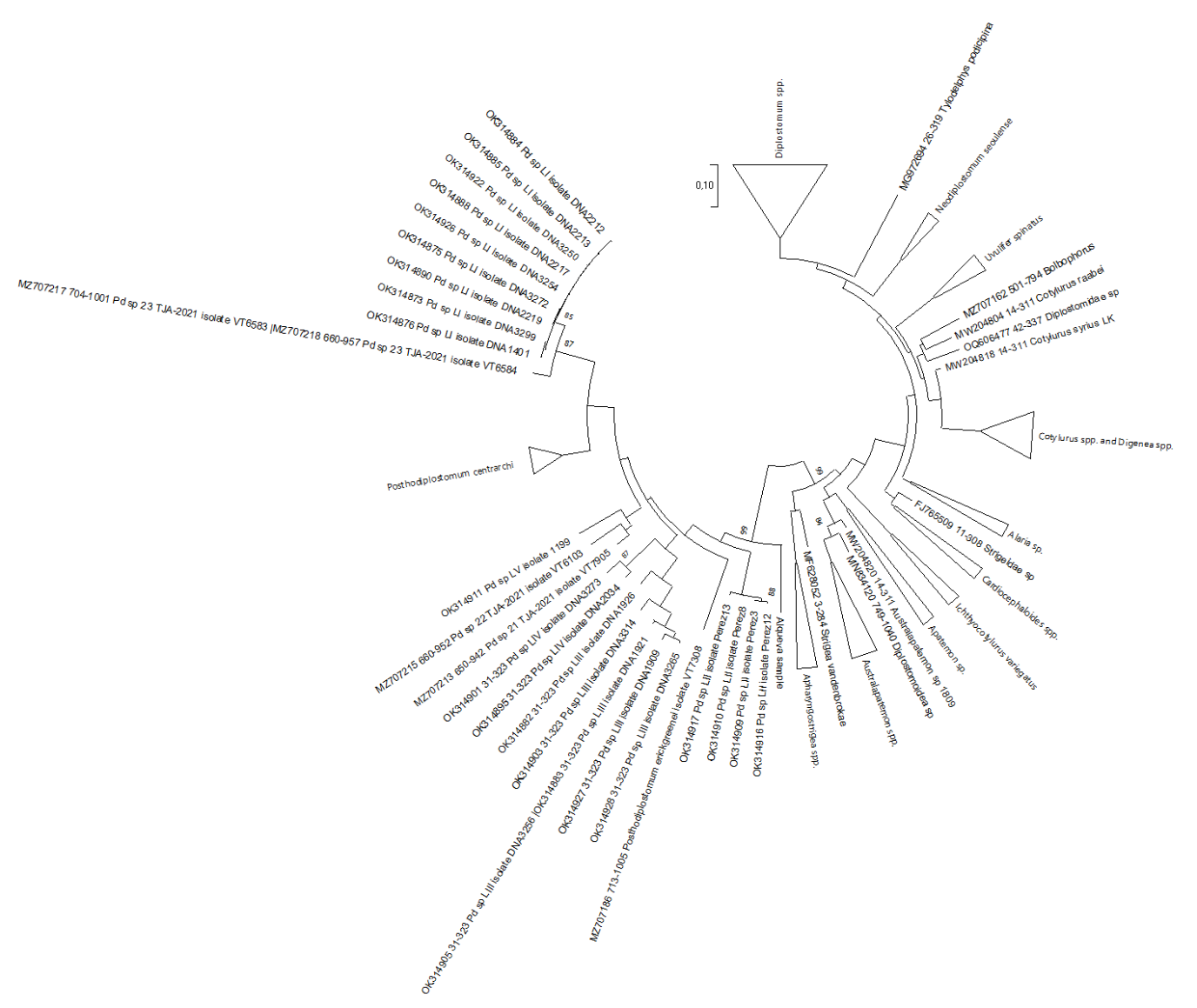

Supplement: Supplementary file 1 [file pathogens-14-00304-s001.zip › Supplementary figures/S3.tif]

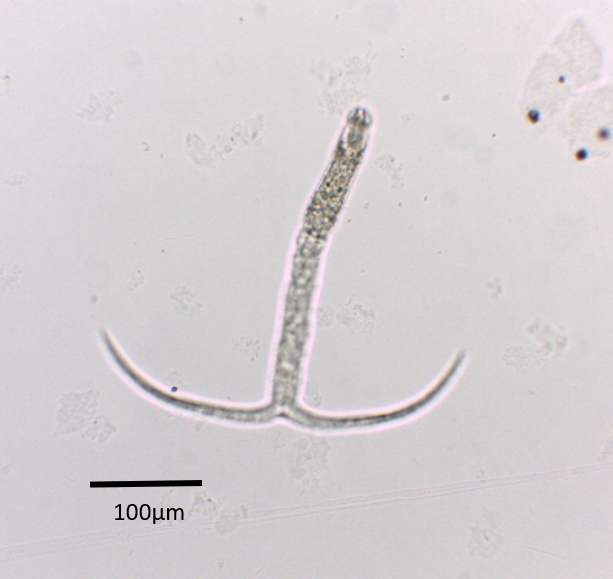

Supplement: Supplementary file 1 [file pathogens-14-00304-s001.zip › Supplementary figures/S4.png]
